# Oxides and Carbonates Accelerate Copper Instability in CO_2_ Electroreduction

**DOI:** 10.1021/jacs.5c21287

**Published:** 2026-02-24

**Authors:** Petru P. Albertini, Saltanat Toleukhanova, Jan Vavra, Anna Loiudice, Vasiliki Tileli, Raffaella Buonsanti

**Affiliations:** 1 Laboratory of Nanochemistry for Energy (LNCE), Institute of Chemical Sciences and Engineering (ISIC), École Polytechnique Fédérale de Lausanne, Sion CH-1950, Switzerland; 2 Institute of Materials, École Polytechnique Fédérale de Lausanne, Lausanne CH-1015, Switzerland

## Abstract

The electrochemical CO_2_ reduction reaction (CO_2_RR) is one of the key chemical transformations promoting the transition from fossil fuel-based energy systems to renewable systems. Copper (Cu)-based materials uniquely catalyze the production of multicarbon (C_2_^+^) products from CO_2_. Yet, copper operational instability limits long-term performance. Herein, we investigate the impact of the chemical nature of the initial Cu surface, particularly oxidation state and carbonate formation, on the structural and operational stability of Cu catalysts along with the reconstruction kinetics of the catalyst. We combine state-of-the-art well-defined catalysts with quasi-operando electrochemical liquid-phase transmission electron microscopy (ec-LPTEM) along with electrochemical characterization to learn about underlying differences. We demonstrate that catalysts with higher initial oxide content undergo faster structural reconstruction and suffer from faster operational deactivation. Interestingly, we find that Cu carbonates further exacerbate structural instability while also suppressing the CO_2_RR activity. Our results highlight the critical role of oxides and carbonates in dictating the reconstruction pathways and durability of Cu under CO_2_RR conditions, offering insights into tuning the Cu-based catalyst design for enhanced CO_2_RR stability and efficiency.

## Graphical abstract

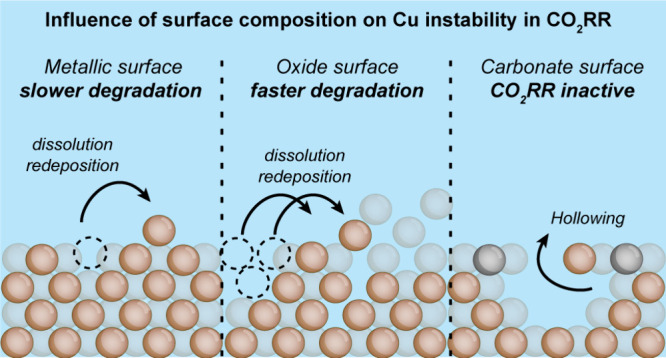

## Introduction

The electrochemical CO_2_ reduction reaction (CO_2_RR) into valuable chemical feedstocks is a promising lever to move from fossil to renewable energy while closing the carbon loop.^1,2^ Cu-based materials remain the most promising catalysts to convert CO_2_ into C_2+_ products, including ethylene, ethanol, and acetate.^3–5^ Efforts have been primarily devoted to catalyst design and cell engineering to overcome the intrinsic lack of selectivity of Cu catalysts, leading to near unity faradaic efficiency for some of the major products at industrially relevant currents.^6^ The overarching hurdle is now the long-term operation stability of CO_2_RR, which the structural reconstruction of Cu-based catalysts substantially contributes to along with electrochemical reactor failure (e.g., flooding).^5,7–9^

Different processes are involved in the reconstruction of Cu catalysts during CO_2_RR.^7,10–12^ First, the Cu surface oxidizes, when starting with metallic surfaces, and dissolves in contact with the aqueous electrolyte at open circuit potential, releasing Cu ions.^10,13^ Then, the oxide reduces and the Cu ions are electrodeposited during the cathodic ramp to operational potential, triggering the first phase of structural reconstruction.^13–16^ Finally, transient carbonyl intermediates drive the second phase of copper reconstruction through surface diffusion and dissolution-redeposition cycles during operational CO_2_RR.^11,12,17,18^ These processes are intrinsic to Cu catalysts for CO_2_RR and, thus, are expected to be independent of the electrochemical reactor used. Yet, discrepancy exists in the literature regarding the operational stability of Cu electrodes, with time scales ranging from less than 1 h to hundreds of hours.^6–8^

While often generically referred to as “Cu electrodes”, the chemical nature of the initial Cu catalyst surface might vary. Indeed, metallic Cu, Cu oxides, and Cu exposed to preconditioning treatments (e.g., exposure to open-circuit potential, air oxidation during electrode preparation) are used across the literature.^10,19–21^ In addition, carbonates have been recently suggested to contribute to copper surface diffusion and associated with reactor failure.^22,23^ The discrepancies in stability of Cu electrodes across the literature suggest that the chemical nature of the initial copper surface might influence reconstruction kinetics and pathways and, thus, operational stability. The impact of the copper oxide on selectivity has been studied.^24,25^ However, studies addressing the impact of the chemical nature of the initial surface on copper reconstruction and stability have been limited so far.^20,24^

Here, we combine well-defined Cu catalysts with quasi-operando electrochemical liquid-phase transmission electron microscopy (ec-LPTEM) along with electrocatalytic performance and electrochemical descriptors to correlate the initial Cu surface composition to the structural and operational stability of Cu catalysts during the CO_2_RR. We demonstrate that oxide-rich catalysts undergo faster structural reconstruction and suffer from faster operational deactivation compared to metallic-rich catalysts. Interestingly, we find that Cu carbonates form at open-circuit potential and upon air exposure and further exacerbate the structural instability while suppressing the CO_2_RR activity, thus providing additional insight into the critical role of carbonates for Cu catalyst deactivation.

## Results and Discussion

### Tuning and Characterizing the Initial Surface Composition of Copper Catalysts

We employed well-defined Cu-based nanocrystals (NCs) as our catalytic platform. We compare metallic Cu cubic NCs, Cu oxide (Cu_2_O) cubic NCs, and pretreated Cu cubic NCs. We choose exposure to open-circuit potential (OCP) and air exposure as pretreatments; we refer to these samples as Cu_OCP_ and Cu_air_ NCs, respectively.

Metallic Cu cubic NCs have been critical for elucidating facet-dependent selectivity,^26,27^ for enabling active site engineering,^28,29^ and for investigating structural degradation during CO_2_RR^18,30^ while being compatible with industrially relevant conditions.^31,32^ Cu_2_O cubic NCs have been widely used to prove the impact of oxides on selectivity.^33,34^ All Cu electrodes eventually undergo exposure to air and open-circuit potential, which motivated us to select these pretreatments. We choose long exposure times for both OCP (15 h) and air (1 week) to mimic day on/day off intermittent operation and an eventual sample handling during shipping/storage, respectively, along with aiming at maximizing the effect of these pretreatments on the sample composition (Figure S1).

We thoroughly characterized the samples by combining different techniques (Figure 1). High-angle annular dark field scanning transmission electron microscopy (HAADF STEM) shows that all catalysts are cubic and have similar size (Figure 1a,e,i,m). More interestingly, the images provide information on the presence and uniformity of surface oxide layers for Cu, Cu_OCP_, and Cu_air_ NCs. The Cu NCs have a lower contrast patchy shell on their surface (Figure 1a–d). High-resolution (HR)­STEM imaging identifies lattice fringes corresponding to Cu_2_O {111} planes (Figure 1b). The Cu_2_O NCs match what is expected for these materials, which is fully crystalline Cu_2_O (Figure 1e–h). The Cu_OCP_ NCs and Cu_air_ NCs both possess a uniform Cu_2_O surface layer (Figure 1i–l,m–p, respectively). The oxide layer is thinner for the Cu_OCP_ (≈1.5 nm) than for the Cu_air_ NCs (≈2.5 nm) based on HRSTEM (Figure 1j,n). Selected area electron diffraction (SAED) (Figure 1q) and electron energy loss spectroscopy (EELS) (Figure 1r) of all samples confirm the oxide identification made via HRSTEM.

**Figure 1:**
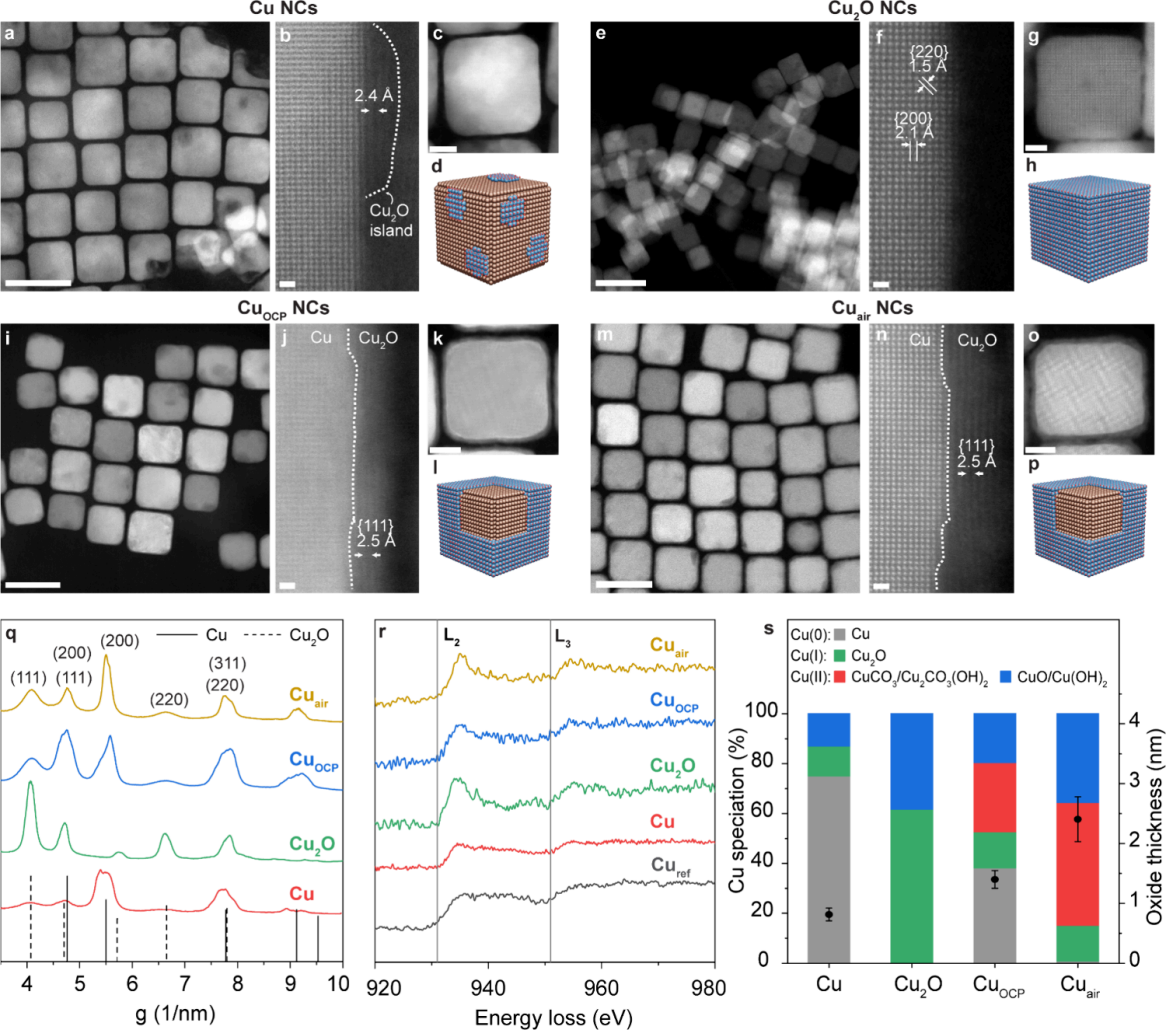
Characterization of Cu catalysts with tunable surface composition. (a–p) HAADF STEM and HRSTEM image along with 3D schematic representation of Cu (a–d), Cu_2_O (e–h), Cu_OCP_ (i–j), and Cu_air_ (m–p) NCs. Scale bars, 50 nm, 10 nm, and 5 Å for HAADF STEM, inset, and HRSTEM images, respectively. (q) SAED radial profile of all studied samples along with references. (r) EEL spectra of the Cu L_3,2_ edge for all studied samples. (s) Surface copper speciation retrieved from X-ray Auger electron spectroscopy (XAES) (left axis) along with the oxide thickness measured from the HRSTEM (b,j,n) (right axis).

Complementary to electron microscopy, Cu LMM X-ray Auger electron spectroscopy (XAES) provides insight into the surface composition (≈5 nm) of the different catalytic systems (Figure 1s and Figure S2). The Cu NCs are predominately metallic with 75% Cu(0), 12% Cu­(I) (Cu_2_O), and 13% Cu­(II) (CuO/Cu­(OH)_2_). Cu_2_O NCs are composed of 60% Cu­(I) and 40% Cu­(II), which is in line with previous studies.^20,35^ The Cu_OCP_ NCs consist of 40% Cu(0) and show a higher fraction of oxide compared with the metallic Cu NCs (Cu­(I) 15% and Cu­(II) oxide 20%). Interestingly, a significant contribution of Cu carbonates (i.e., CuCO_3_ and Cu_2_CO_3_OH) emerges (c.a. 25%) for this sample. The Cu_air_ NCs have only traces left of metallic Cu (less than 1%) and consist of Cu oxide (Cu­(I) oxide 15% and Cu­(II) oxide 35%) and Cu carbonate (c.a. 50%). Studies on Cu atmospheric corrosion report on the formation of Cu carbonate.^36,37^ The data reported explicitely correlate the formation of Cu carbonate and CO_2_RR conditioning. Specifically, the formation of Cu carbonate depends on the presence of CO_2_ and on the time of exposure to air (Figure S3).

### Catalytic Performance for CO_2_RR

Next, we evaluated the impact of the initial surface composition of the Cu catalysts on the catalytic performance for the CO_2_RR (Figure 2 and Table S1 and Figure S4). We focused on −1.1 V vs RHE as one representative potential comparable with previous studies.^24–29,33–35^

**Figure 2:**
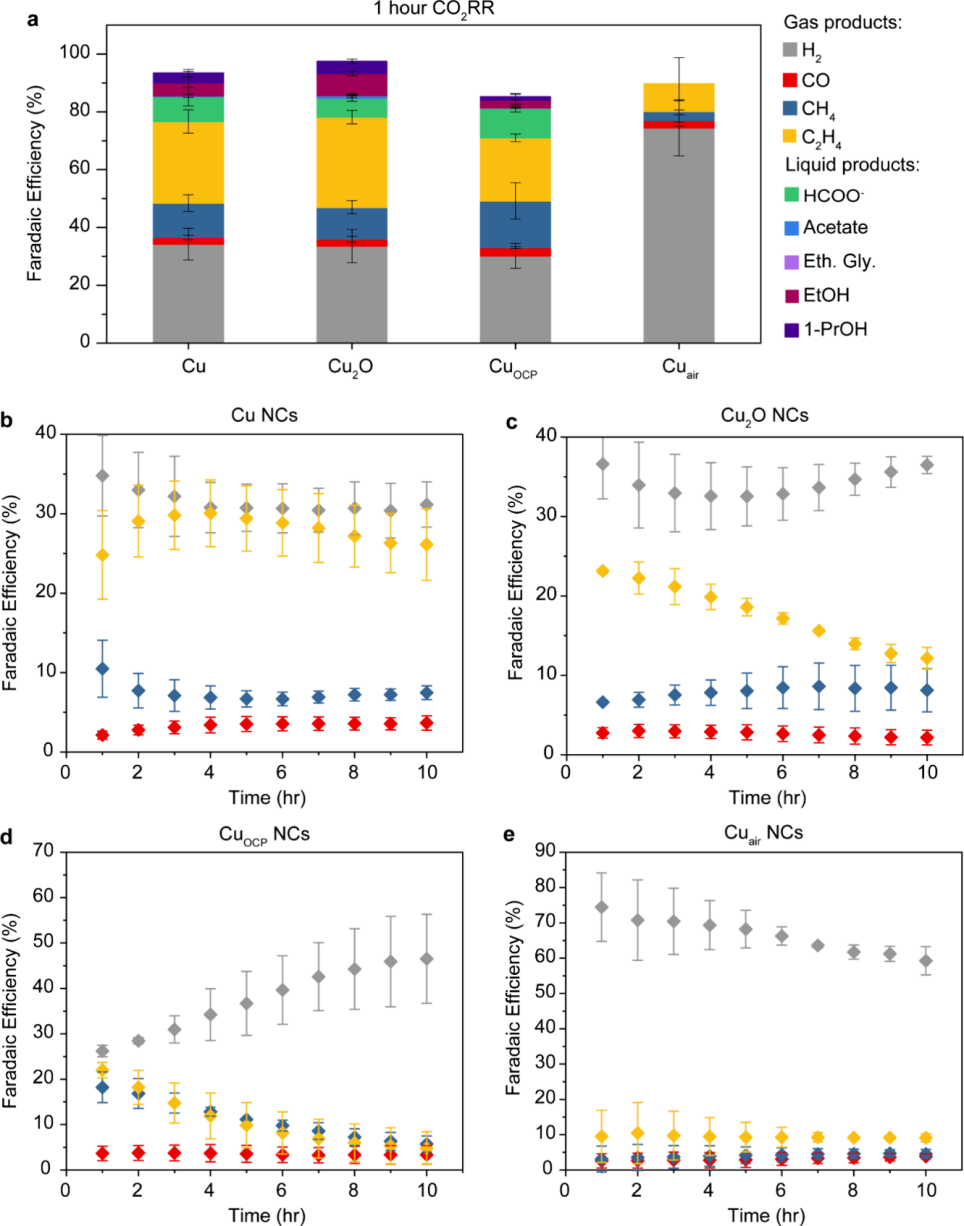
CO_2_RR performance and long-term operational stability. (a) Total FEs for all gaseous products (that is, H_2_, CO, CH_4_, and C_2_H_4_) and the main liquid products (that is, HCOO^–^, acetate, ethylene glycol, C_2_H_5_OH, and 1-propanol) at −1.1 V vs RHE for 1h. (b–e) Evolution of the FEs for all gaseous products (that is, H_2_, CO, CH_4_, and C_2_H_4_) during 10 h of CO_2_RR for Cu (b), Cu_2_O (c), Cu_OCP_ (d), and Cu_air_ (e) NCs. The data are the average of three independent experiments, and the error bars are the calculated standard deviation.

First, we comment on the faradaic efficiency (FE) of each sample, which was calculated based on the first hour of operation (Figure 2a). The Cu and Cu_2_O NCs are mostly selective for ethylene with around 30% faradaic efficiency (FE). The results are in line with previous studies.^24–29,34,35^ Interestingly, the FE for methane of Cu_OCP_ NCs is nearly double compared to Cu NCs (from around 10% to 20%) while the FE of ethylene drops to 20%. Finally, Cu_air_ NCs are mostly selective for hydrogen with an FE above 60%.

Second, differences between the catalysts become more pronounced when looking at the FE over the 10 h CO_2_RR (Figure 2b–e, Table S1 and Figure S4). The product distribution of the Cu NCs remains mostly stable compared with the other samples (Figure 2b). A slight decay in ethylene FE occurs starting at around 6 h, matching our previous results.^30^ The Cu_2_O NCs show signs of earlier CO_2_RR deactivation (Figure 2c); here, ethylene FE decreases after 2–3 h. This observation is consistent with previous studies.^25,33,34^ Interestingly, the Cu NCs oxidized to match the surface composition of Cu_2_O NCs also show an earlier sign of CO_2_RR deactivation (Figure S5). This observation strengthens the connection between the higher oxide content and accelerated CO_2_RR deactivation. A more pronounced HER increase at the expense of the CO_2_RR occurs for Cu_OCP_ (Figure 2d). This faster loss in ethylene selectivity for Cu_OCP_ compared to Cu_2_O and Cu_H2O2_ indicates that the Cu speciation strongly influences the catalyst deactivation rates, where the presence of Cu carbonate seems to accelerate those effects. Cu_air_ remains almost inactive for CO_2_RR over the 10 h with more than 60% hydrogen FE (Figure 2e).

Altogether, these observations indicate a clear difference in the stability of the product distribution of the investigated systems, which was herein compared under the same conditions for the first time. We observed comparable, if not poorer, C_2+_ selectivity between Cu and the oxide-rich catalysts. Our data highlight that oxide-derived catalysts do not necessarily enhance C_2+_ selectivity but rather greatly impact the operational stability of the catalyst (Supplementary Note 1). All the oxide-rich catalysts undergo faster operational deactivation for ethylene selectivity compared to the metallic-rich catalyst while high content of surface carbonates generate a HER-active Cu catalyst.

### Quasi-Operando Liquid-Phase TEM Monitoring Catalyst Restructuring

We chose electrochemical liquid-phase transmission electron microscopy (ec-LPTEM) to follow the eventual restructuring of the NCs triggered by the different surface composition. ec-LPTEM has been demonstrated to provide valuable insight into Cu catalyst reconstruction during the initial stages of CO_2_RR.^13,14,17,30,34,38^ While ec-LPTEM operates on a short-time scale, the structural changes monitored by this technique have been essential to better understand CO_2_RR catalyst behavior on longer time scales.^13,14,17,30,34,38^ In contrast, insights gained from post-mortem TEM have been less useful because of the structural and compositional Cu changes occurring upon exposure to OCP.^10,14,39–41^

We monitored the morphological and structural changes during the potential ramp from OCP to −0.8 V vs RHE by linear sweep voltammetry (LSV) (i.e., start-up) and subsequent chronoamperometry (CA) at −0.8 V vs RHE for 5 min via synchronized image sequences, particle analysis data, and selected area electron diffraction (SAED) (Figure 3) along with performing several control experiments, including the exclusion of beam effects (Figures S6–S12).

**Figure 3:**
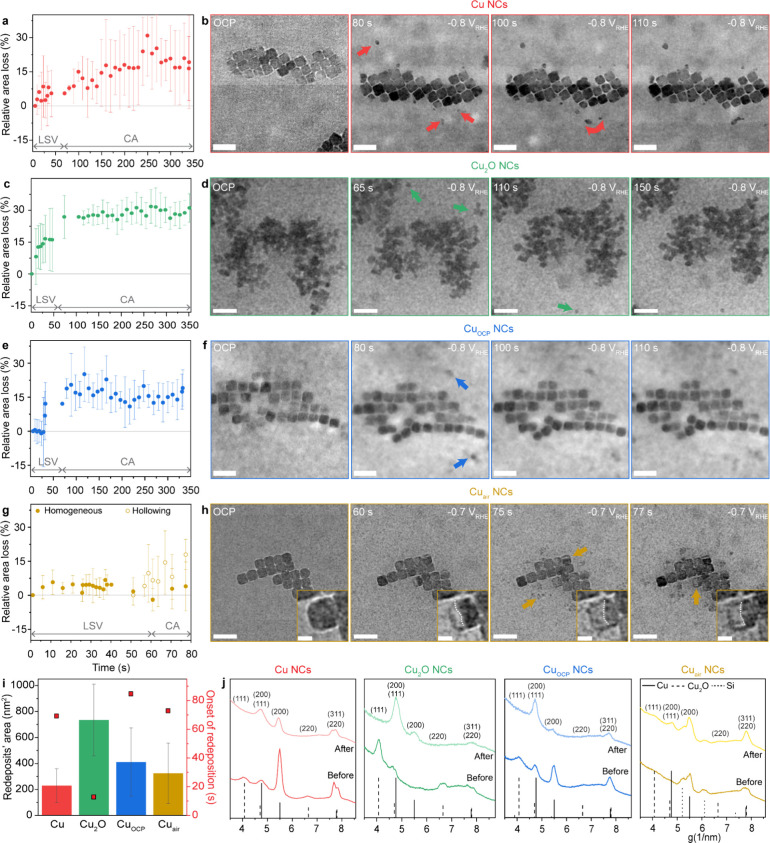
Quasi-operando liquid-phase TEM monitoring catalyst restructuring at CO_2_RR conditions. (a,c,e,g) Relative area loss of Cu (a, red), Cu_2_O (c, green), Cu_OCP_ (e, blue), and Cu_air_ (g, yellow). (b,d,f,h) Time-lapse images of Cu (b), Cu_2_O (d), Cu_OCP_ (f), and Cu_air_ (h) NCs during LSV-CA. Arrows of corresponding color indicate redeposited particles in panels (b,d,f). Arrows in panel (h) indicate hollowing events of primary Cu NCs. Scale bar: 100 nm. In panel (h), insets depict hollowing of one single NC. Scale bar, 20 nm. (i) Average area of redeposited particles and the onset time of redeposition for all samples. (j) *In situ* SAED of NCs before and after LSV-CA measurement.

The Cu NCs experience a progressive decrease in the projected area during the LSV, which continues during the CA up to 250 s before stabilizing at a total relative area loss of around 15% (Figure 3a). The synchronized image sequence shows that the formation of secondary particles (at ca. 70s) accompanies the size decrease of the Cu NCs (Figure 3b, Figures S6 and S7). The behavior of the Cu NCs agrees with previous *ex situ* observations on the same NCs.^30^ The size decrease and formation of the secondary particles is indicative of the dissolution-redeposition process driving Cu reconstruction.^13,14,18^

The Cu_2_O NCs undergo a more pronounced and steeper decrease in the projected area during LSV compared with the Cu NCs. The change continues during the first 30 s of CA before stabilizing at a total area loss of around 30% (Figure 3b). Concomitantly, an earlier formation onset of secondary particles (at ca. 10 s) and significant sintering of the Cu_2_O NCs occurs (Figure 3c,d and Figure S8). These observations are in line with previous *ex situ* and *in situ* results on the same NCs.^33,34^

The Cu_OCP_ NCs exhibit a similar behavior to the Cu NCs, although with most change occurring during LSV and less change occurring during CA with fewer secondary particles forming (Figure 3e,f and Figure S9).

The Cu_air_ NCs differ significantly in their restructuring behavior from the rest of the samples with their unique facet-selective core etching (hollowing) and a shell (edge and corner) remaining intact while secondary particles still form (Figure 3g,h).

Plotting the average area of redeposited secondary particles together with the onset time of their observation indicates a faster and more drastic reconstruction occurring for the Cu_2_O NCs among all samples and a larger extent of reconstruction for the Cu_OCP_/Cu_air_ compared to the Cu NCs (Figure 3i).

Complementary, *in situ* SAED of the samples confirms that all the Cu catalysts consist of mostly a metallic phase after LSV-CA (Figure 3j).

Strikingly, stabilizing the Cu surface by means of an inert oxide shell (here amorphous ZrOx) following our previous studies results in no change in the ec-LPTEM (Figure S10–S12), which confirm the importance of redox surface changes in the Cu stability during CO_2_RR.^29,42,43^

Overall, the quasi-operando electron microscopy evidence different kinetics among the CO_2_RR active Cu samples (i.e., Cu, Cu_2_O, and Cu_OCP_) while a different mechanism emerges for the Cu_air_, which is HER-active.

### Electrochemical Characterization Monitoring Catalyst Active Area and Selectivity

Additionally, we examined in more depth the partial current densities, the electrochemically active surface area (ECSA), and the selectivity trends from the benchtop measurements (Figure 4).

**Figure 4:**
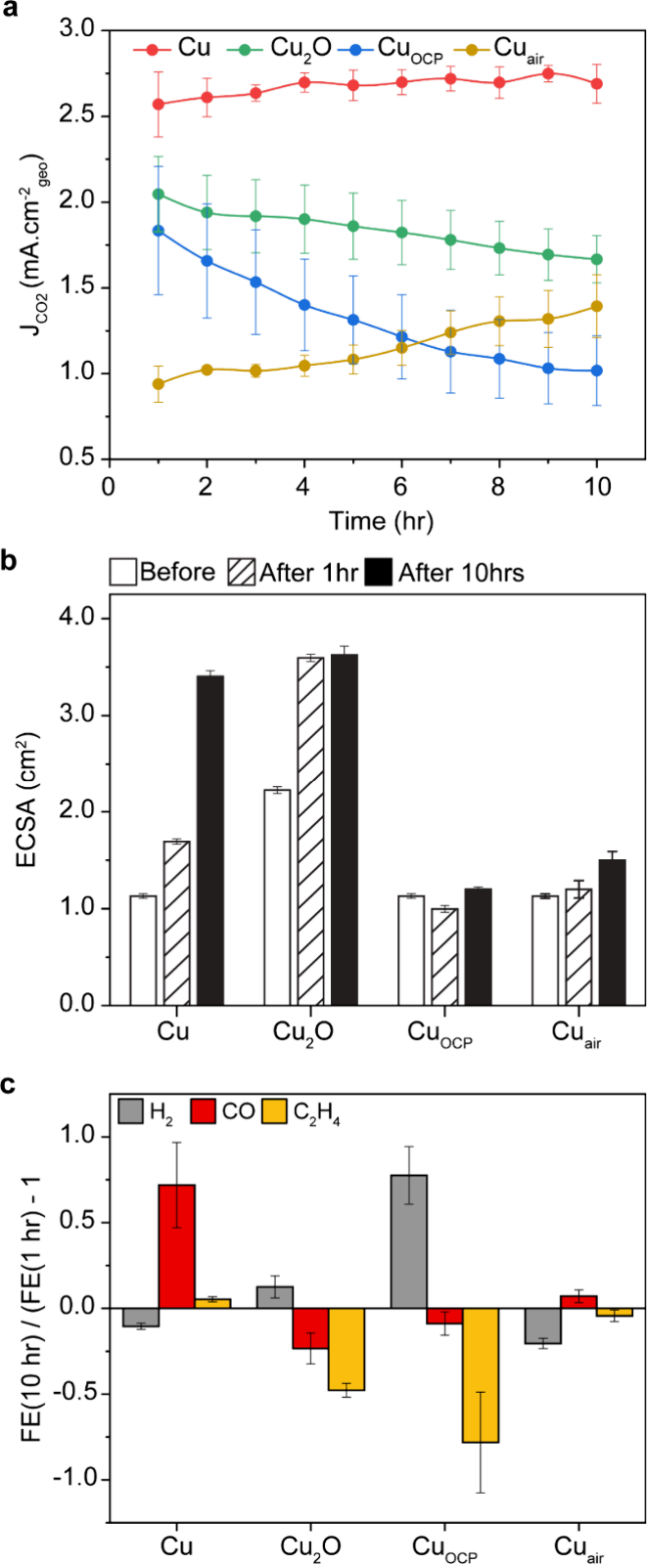
Electrochemical characterization monitoring catalyst active area and selectivity. (a) CO_2_ partial current density normalized by the geometric area as a function of time. (b) ECSAs from Pb-UPD of the different catalysts before CO_2_RR and after 1 and 10 h of CO_2_RR 5 min at −1.1 V vs RHE in CO_2_RR conditions were applied on Cu_2_O prior Pb-UPD for “before”. (c) Relative change in selectivity for H_2_, CO and C_2_H_4_ between 1 and 10 h.

The change in the CO_2_ partial current density normalized by the geometric surface area (J_CO2‑geo_) as a function of time reflects the evolution of the total number of CO_2_RR active sites during the 10 h electrolysis (Figure 4a). The data indicate that Cu_2_O and Cu_OCP_ NCs undergo the most drastic change. Indeed, the J_CO2‑geo_ values of the Cu NCs remain stable. On the contrary, the J_CO2‑geo_ of the Cu_2_O NCs and Cu_OCP_ NCs decreases over time, with a final loss of 20 and 45%, respectively. Cu_OCP_ NCs deactivate faster than the Cu_2_O NCs. The Cu_air_ NCs remain overall inactive for the CO_2_RR during the 10 h. The total current density also decreases for the Cu_2_O NCs and Cu_OCP_ NCs without any catalyst detachment being observed (Figure S13). Additionally, the J_CO2‑geo_ of surface-oxidized Cu NCs, which possess similar Cu(0) content of Cu_OCP_ NCs yet no carbonate (Figure S5), decreases similarly to Cu_2_O NCs (Figure S14), which points to carbonates playing a key role in deactivation kinetics.

The observed evolution over time in current density, while knowing that all catalysts reconstruct, indicate that the number of active sites and/or their selectivity (e.g., more sites that are less active or active for different products) must change for all.

We used ECSA to qualitatively examine the change in the total number of active sites, whether CO_2_RR or HER-active sites (Figure 4b and Figure S15). The ECSA of the Cu NCs continuously increases from before the CO_2_RR to 10 h after CO2RR. The ECSA of the Cu_2_O NCs increases from before to 1 h to reach the same value of the Cu NCs. The ECSA of Cu_OCP_ and Cu_air_ NCs only slightly fluctuates around a value that is lower compared to the Cu NCs and Cu_2_O NCs. Pb-UPD confirms the slower transformation of the Cu NCs from low-coordinated atoms (i.e., defects, corners (111), and edges (110)) to more stable coordination geometry (100) compared to the Cu_2_O NCs (Figure S16,17).

Then, we looked at the relative change in selectivity for hydrogen, CO, and ethylene between 1 and 10 h of the CO_2_RR (Figure 4c). In addition to hydrogen and ethylene, we chose CO because of its role as key intermediate in the CO_2_RR mechanism and in driving copper reconstruction via Cu-CO intermediate formation.^7,11,12,15,18,44^ The ethylene remains constant for Cu NCs while it decreases for Cu_2_O and Cu_OCP_ NCs. Hydrogen increases for Cu_2_O and Cu_OCP_ NCs. The CO selectivity increases only for Cu NCs. Cu_air_ does not undergo major change.

## Discussion

The evolution of the catalytic performance and the catalyst restructuring indicate a clear difference between the Cu, Cu_2_O, Cu_OCP_, and Cu_air_ catalysts, which possess a different initial surface composition. The presence of oxides and carbonates correlates with faster operational CO_2_RR deactivation and structural changes to the extreme point of having a catalyst not active at all for CO_2_RR (i.e., Cu_air_). Interestingly, previous studies in flow cells and MEA also point at a higher stability of metal Cu compared to Cu oxide-derived catalysts.^7,31,32,34,45,46^

Figure 5 provides a summary of the findings. The catalysts with higher initial oxide content (i.e., Cu_2_O followed by Cu_OCP_) undergo faster and/or more drastic structural changes compared to catalysts with higher initial metallic content (i.e., Cu NCs). The metal-rich Cu exhibits a more stable CO_2_RR and, particularly, C_2+_ selectivity compared to oxide-rich Cu. Eventually, CO selectivity increases for the metal-rich Cu while hydrogen selectivity increases for oxide-rich Cu. Remarkably, the CO_2_RR deactivation effect becomes even more pronounced in the presence of Cu carbonates and HER dominates along with a uniquely different reconstruction pathway (i.e., Cu_air_ NCs).

**Figure 5:**
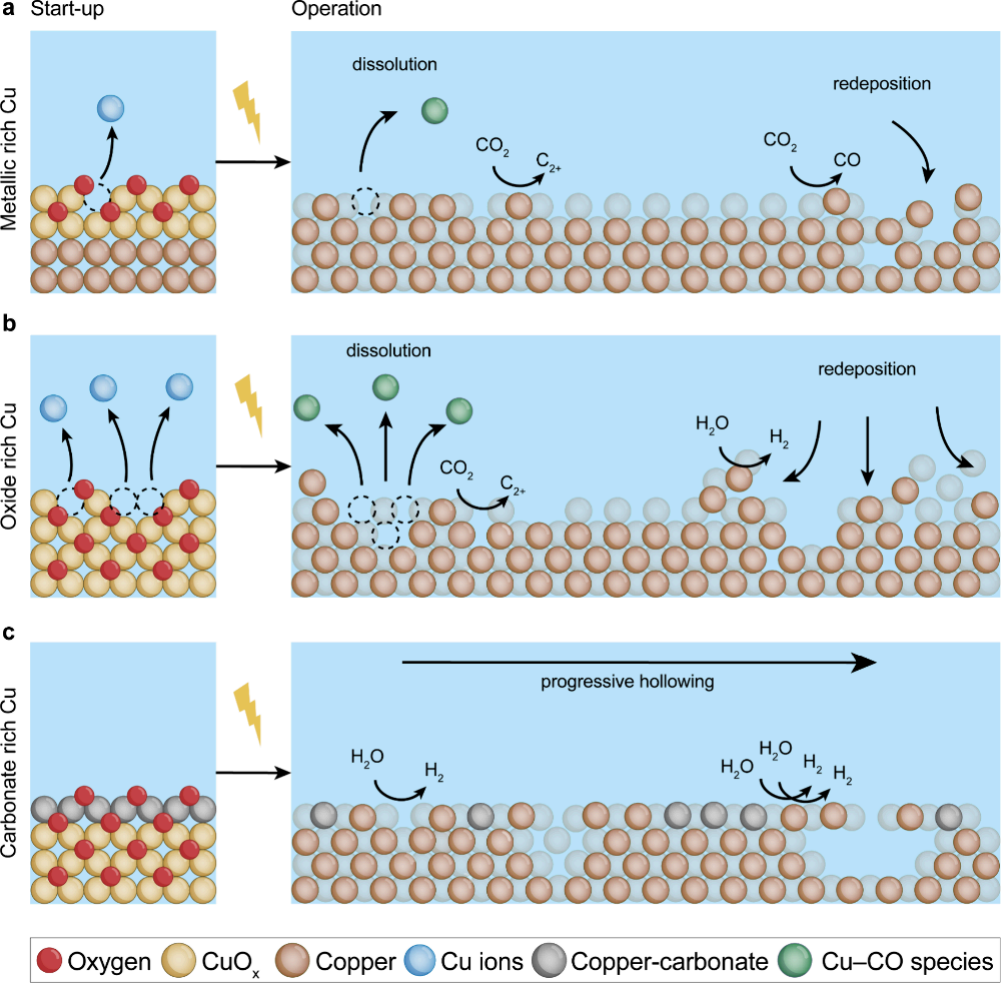
Schematic representation summarizing the impact of the initial Cu catalyst composition on catalyst reconstruction and the CO_2_RR selectivity.

Metallic Cu dominates in all catalysts at cathodic potential, corroborated here by the quasi-operando ec-LPTEM and in agreement with previous studies.^14,21,25,35,42,47^ The data suggest that the initial surface Cu composition impacts the reconstruction kinetics and the type of generated active sites responsible for selectivity.

Studies evidence that the greater the initial oxide content on copper is, the more the formation of grain boundaries, defects, and undercoordinated atoms during CO_2_RR is favored.^14,15,18,24,40,41,48–50^ A few studies indicate that these low-coordinated sites interact more weakly with CO.^51^ We suggest that the oxide-rich Cu transforms more rapidly into these low-coordinated sites, shifting the selectivity from the CO_2_RR to the HER-active sites. The observation of continuously increasing CO selectivity for the metal-rich Cu might indicate that different low-coordinated active sites are generated from the slower reconstruction or that the slow generation of the same weakly CO-interacting low-coordinated active sites enables the CO detection. HER might then still take over at longer operation time than those studied in this work.

A few reports hint at poor CO_2_RR activity associated with Cu carbonates.^14,52^ Postelectrolysis Cu oxide cubes formed at OCP in carbonate electrolyte showed irreversible structural transformation and suppressed C_2+_ selectivity compared to the initial Cu spheres, hinting at a possible detrimental role of Cu carbonate.^14^ One study demonstrated that Cu carbonate is a poor CO_2_RR catalyst compared to Cu oxide-derived catalysts.^52^ Complementary *in situ* surface-enhanced Raman spectroscopy (SERS) indicates persistence of the Cu-carbonate signal during the CO_2_RR (Figure S18andTable S2). Thus, we suggest that the Cu carbonate might render the surface inaccessible for CO_2_RR or that the reduction of Cu carbonates generates active sites with poor binding affinity for CO_2_RR intermediates binding affinity.

As for the observed hollowing for the carbonate-rich Cu, competing reconstruction mechanisms might occur in the absence of significant CO_2_RR activity, which are different than the CO-driven reconstruction.^7^ In particular, cathodic corrosion has been previously identified to drive reconstruction of HER-active catalysts and has been recently suggested to cause hollowing of Cu cubes, although not yet connected to carbonate presence and HER activity.^53–55^ One alternative or contributing pathway might involve continuous reduction–oxidation cycles of surface Cu wherein the reduction is voltage-driven while the reoxidation is caused by OH* radicals generated from oxygen transfer between H_2_O and HCO_3_^–^.^56^ This cycle might ultimately lead to the dissolution of the internal metallic copper of carbonate-derived Cu NCs through hollowing.

## Conclusions

In conclusion, this study clarifies how the initial copper surface composition critically dictates structural catalyst evolution and operational stability during the CO_2_RR. Our results underscore that greater initial oxide content triggers faster reconstruction and CO_2_RR performance degradation. Interestingly, the formation of Cu carbonates emerges as particularly detrimental, potentially persisting under cathodic conditions and promoting irreversible surface changes promoting HER and new reconstruction pathways. These findings might help to explain previously conflicting literature and emphasize the importance of precise control over surface composition and preconditioning in designing robust Cu catalysts. The comparison of Cu catalysts with different initial copper surface composition under the same conditions also raises an interesting question on the differences in their product selectivity, which might open up follow up studies.

Overall, this work inspires future strategies that consider both chemical and structural factors in catalyst preparation to achieve selective and durable CO_2_RR performance across various reactor platforms.

## Supplementary Material



## Data Availability

Experimental data are openly available in Zenodo at https://zenodo.org/uploads/18235845

## Notes

Supplementary methods; additional experimental considerations; supplementary notes; additional TEM, XPS, Pb-UPD, and CO_2_RR (PDF)
