# Supplementary material for: Oxides and Carbonates Accelerate Copper Instability in CO2 Electroreduction
Source: J Am Chem Soc. 2026 Feb 24;148(9):9784–92. doi: 10.1021/jacs.5c21287 (PMC12983321; doi:10.1021/jacs.5c21287)
Supplement: Supplementary file 1 [file ja5c21287_si_001.pdf]

Supplementary Information

Oxides and carbonates accelerate copper instability in CO_2 electroreduction

Petru P. Albertini[1,+], Saltanat Toleukhanova[2,+], Jan Vavra[1,2], Anna Loiudice[1],Vasiliki Tileli[2*], Raffaella Buonsanti[1*]

[1]Laboratory of Nanochemistry for Energy (LNCE), Institute of Chemical Sciences and Engineering (ISIC), École Polytechnique Fédérale de Lausanne, CH-1950 Sion, Switzerland.

[2]Institute of Materials, École Polytechnique Fédérale de Lausanne, Lausanne CH–1015, Switzerland

[+] these authors contributed equally

* Corresponding author: vasiliki.tileli@epfl.ch and raffaella.buonsanti@epfl.ch

Table of Contents

Synthetic Methods 3
Material Characterization 5
Electrochemical Liquid-Phase TEM (ec-LPTEM) Experiments 5
Electrochemical Characterization and Electrocatalytic Measurements 8
In-situ surface-enhanced Raman spectroscopy (SERS) 11
Figure S1. STEM EELS analysis of Cu NCs at different time of OCP exposure. 12
Figure S2. Cu LMM X-ray Auger electron spectroscopy (XAES) 13
Figure S3. Cu surface speciation from LMM X-ray Auger electron spectroscopy (XAES). 14
Table S1. Faradaic Efficiency 15
Figure S4. Total FEs for all gaseous products 16
Figure S5. Surface composition and CO_2RR performances of surface oxidized Cu NCs. 17
Supplementary Note 1: 18
Figure S6. ec-LPTEM control experiments without applied potential. 19
Figure S7. TEM characterization of Cu NCs before and after ec-LPTEM during CO_2RR. 20
Figure S8. TEM characterization of Cu_2O NCs before and after ec-LPTEM during CO_2RR. 21
Figure S9. TEM characterization of Cu_{OCP} NCs before and after ec-LPTEM during CO_2RR. 22
Figure S10. Characterization of Cu@ZrO_x NCs catalyst. 24
Figure S11. EELS analysis of Cu@ZrOx NCs and bulk ZrO_x. 25
Figure S12. ec-LPTEM experiments on Cu@ZrO_x NCs at CO_2RR relevant conditions. 26
Figure S13. Total current density over 10 h of CO_2RR. 27
Figure S14. Evolution of the CO_2 partial current density normalized by the geometric area as a function of time 28
Figure S15. Representative Pb underpotential deposition before and after 1 and 10 h CO_2RR 29
Figure S16. Facets distribution from Pb-UPD. 30
Figure S17. Facet distribution for Pb-UPD for Cu and Cu_2O NCs before and after 1 and 10 h of CO_2RR. 31
Figure S18. In-situ surface-enhanced Raman spectroscopy (SERS) of Cu and Cu_{OCP} 32
References 36

Synthetic Methods

Materials

Copper(I) bromide (CuBr, 90.999%), oleylamine (OLAM, 70%), oleic acid (OLAC, technical grade, 90%), Tri-n-octylphosphine oxide (TOPO, 99%), octane (OT, anhydrous, ≥99%), toluene (anhydrous, 99.8%), ethanol (EtOH, anhydrous, 95%), hydrogen peroxide (H_2O_2, 50 % (w/w) in H_2O, contains stabilizer) and Nitric acid (HNO_3, 70%, purified by re-distillation, ≥99.999% trace metals basis) were purchased from Sigma Aldrich. Hexane (anhydrous, >96%) was purchased from TCI Deutschland GmbH and tetrakisdimethylamidozirconium (98%, 99.99% Zr) were purchased from ABCR Swiss AG. OLAC was degassed by stirring under dynamic vacuum at 130 °C for a 1-hour before storing in an N_2-filled glove box.

Synthetic Methods

General Consideration

All syntheses and manipulations of Cu NCs were performed under a dry N_2 atmosphere, using Schlenk-line techniques or a glove box. Anhydrous organic solvents were used for the manipulation, analysis and storage of Cu NCs. All volumes below 20 mL were measured and dispensed using Eppendorf microliter pipettes. All glassware was stored in oven at 110 ºC to minimize air/moisture contamination. Toluene during the oxide growth was dried over activated molecular sieves.

Synthesis of cubic Cu NCs

Cubic Cu NCs were synthesized following a protocol adapted from Iyengar et al.[1] A typical synthesis was conducted in a 250 mL three-necked glass round bottom flask equipped with a straight condenser, a thermocouple in a glass tube, a septum and an olive shape Teflon stirrer. Tri-octyl phosphine oxide (TOPO, 9.37 g, 24.2 mmol) was added to the flask at room temperature and was dried/degassed under vacuum for 30 min. Oleylamine (OLAM, 116 mL) was then added to the flask and the OLAM/TOPO mixture was dried/degassed for 30 min under vacuum at 130 ºC. The flask was then refilled with N_2 gas and cooled to 50 ºC. Cu(I)Br (717 mg, 99.999%, 5 mmol) stored in a N_2 filled Eppendorf was quickly added into the reaction mixture forming a light green solution. The solution was stirred under vacuum for 30 min at 80 ºC to ensure full dissolution of Cu(I)Br while removing the residual oxygen introduced during the Cu(I)Br addition. The solution turned light yellow. After 30 min, the reaction

mixture was rapidly heated to 260 °C, and the flask was refilled with N_2 around 120-130 ºC. Once reaching 260 ºC, the solution rapidly turned red then turbid violet. After 30 min at 260 °C, the colloidal suspension was allowed to cool down to room temperature by removing the heating mantle. Then, the reaction mixture was transferred into four 40 mL glass vials with a septum, filled with N_2, with an inert 20 mL plastic syringe. To each 5 mL portion of the crude reaction mixture, toluene (5 mL) and ethanol (15 mL) were added. Six 50 mL centrifuge tubes were used initially. The particles were isolated by centrifugation at 6000 rpm for 8 minutes, and the supernatant was discarded. The particles were combined into two 50 mL centrifuge tubes then washed using toluene (5 mL) and ethanol (15 mL) and again isolated after centrifugation. Finally, the particles were re-dispersed and combined into a single suspension using 10 mL toluene and were stored in an N_2-filled glovebox. In between the washing steps, sonification may be done to help with NCs re-suspension.

Synthesis of Cu_2O NCs

Cubic Cu NCs were synthesized following a protocol adapted from Zhan et al.[2] In a typical synthesis, 4 ml 0.1 M $CuSO_4$ were stirred vigorously in in 366 ml MilliQ water. Then 14 ml 1 M NaOH were added. The solution turns from pale blue to pale white. After 10 s, 16 ml of 0.25 M L-Ascorbic Acid was added. The solution turns progressively from pale to intense yellow. The solution was stirred further for 13 min. The obtained reaction medium was centrifuged three times with a EtOH:H2O mixture (1:1) and two times with EtOH at 6000 rpm for 8 min each time. The clean sample was stored in 15 ml of EtOH. The Cu concentration was measured by ICP-OES.

Preparation of Cu_{ocp} NCs

The Cu_{OCP} NCs were prepared by drop-casting 30 ug of as-synthesized Cu NCs on a clean glassy carbon (or silicon wafer for XPS) and immersed in 5 mL of 0.1 M KHCO3 constantly purged with 5 mL/min of CO_2 for 15 hours.

Preparation of Cu_{air} NCs

The Cu_{air} NCs were prepared by drop-casting 30 ug of as-synthesized Cu NCs on a clean glassy carbon (or silicon wafer for XPS) and exposed to ambient air for one week.

Material Characterization

X-ray photoelectron spectra (XPS) were recorded using an Axis Supra (Kratos Analytical) instrument, using the monochromated Kα X-ray line of an Al anode. The pass energy was set to 20 eV with a step size of 0.1 eV. The samples were electrically insulated from the sample holder and charges were compensated. Spectra were referenced at 284.8 eV using the C–C bound of the C 1s orbital. Cu NCs samples were prepared by drop-casting nanocrystal films onto clean Si substrates.

Bright-field transmission electron microscopy (TEM) was performed on a Thermo Fisher Scientific Tecnai-Spirit instrument operating at 120 kV. To prepare the sample for imaging, 20- 40 µL of the as-synthesized NPs were drop-casted on a carbon-coated Cu TEM grid (Ted Pella Inc.).

Inductively coupled plasma optical emission spectroscopy (ICP-OES) was performed on an Agilent 5100 instrument equipped with a VistaChip II CCD detector. Typically, 10 µL of NCs suspension were dried and digested overnight in 142.5 µL of 70%v/v nitric acid ICP grade solution (Sigma-Aldrich). For oxide samples (e.g., Cu_2O NCs), 100uL of HCl 37% was added to the nitric acid solution. Milli-Q water was added to obtain a 5mL solution with a 2% acid content solution. Before each analysis, ICP grade standard solutions (1000 ppm in 2% nitric acid) were diluted with 2 wt.% of nitric acid to prepare the calibration curve used for the determination of the sample concentrations.

Electrochemical Liquid-Phase TEM (ec-LPTEM) Experiments

Sample preparation

The pretreated OCP samples involved a dispersion of Cu NCs in toluene (1500 µg/ml) that was drop-casted using a glass capillary needle onto the glassy carbon working electrode of the electrochemical chip (in-house fabricated). The chip was fixed inside the custom chip holder for connection to the potentiostat, then immersed together with a bulk counter (Pt wire) and reference (leakless Ag/AgCl) electrodes in the beaker filled with CO_2-saturated 0.1M $KHCO_3$ electrolyte. Once the system was connected to the potentiostat (Biologic), open circuit conditions were kept for 30 min (Cu_{cub}) and for 15 h (Cu_{OCP}). The electrochemical chip was then collected from the beaker, rinsed with deionized water and dried with air gun.

For the CuO_2 NCs, their dispersion in ethanol (1500 μg/ml) was drop-casted directly on glassy carbon electrode with glass capillary.

The Cu_{air} NCs were prepared by exposing the drop-casted Cu NCs to air.

For in situ TEM experiments, the sample loading was kept low to avoid agglomeration of particles and, thus, to facilitate further analysis of images. Each sample's drop-casted chip was immediately assembled into the liquid cell holder (Hummingbird Scientific) together with the bottom spacer chip and O-rings. Then CO_2-saturated 0.1 M $KHCO_3$ electrolyte was flushed through the fluidic lines to wet the cell, prior to the insertion of thee holder inside the electron microscope (JEOL 2200FS).

For the post-mortem EELS measurements, the chips were then immediately mounted into a single-chip holder (Hummingbird Scientific) and inserted into the electron microscope (Thermo Fisher Scientific Titan Themis 60-300) to avoid extra air exposure.

Electron microscopy and electrochemical measurements

All ec-LPTEM measuremets were performed on a JEOL 2200FS (JEOL Ltd.) equipped with in column Ω-filter and a direct electron camera (Direct Electron) for energy-filtered imaging at a temporal resolution of 20 frames per second. Dedicated liquid cell electrochemical holder (Hummingbird Scientific). The electrochemical top chip was custom-fabricated for CO_2RR experiments and features a patterned planar electrode system composed of a glassy carbon working electrode and platinum counter and reference electrodes. The bottom, spacer chip was 100 nm (Hummingbird Scientific). Biologic SP-300 potentiostat equipped with an ultra-low current cable was used to drive the electrochemical reaction. In situ imaging was done at 200 kV high voltage, using a spot size of 5, alpha 3, condenser lens aperture 2, electron flux rate of 25 - 30 $e^{-}nm^{-2}s^{-1}$, and temporal resolution of 20 frames per second (StreamPix software). Selected area electron diffraction (SAED) was conducted before and after in situ TEM measurement using a selected area aperture 1, a camera length of 800 mm, and an exposure time of 1 second. Both SAED and in situ TEM imaging were performed in energy-filtered mode with an energy-selecting slit width of 10 eV centered on the zero-loss peak.

The in situ SAED measurements were performed by acquiring SAED patterns (exposure time, 1 sec) every two minutes while keeping the sample at OCP. The beam shutter was inserted in

between acquisitions to avoid sample exposure to electron beam. The radial profile of SAED was obtained with Radial Profile plugin in ImageJ software.

Electron energy loss spectroscopy (EELS, Dual-EELS) was done on Titan Themis (Thermo Fisher Scientific) at 300 kV high voltage in scanning TEM (STEM) mode using a 5 mm GIF entrance aperture, a camera length of 400 m, convergence semi-angle 20 mrad, collection semi-angle 56.8 mrad, a dispersion of 0.18 eV/Ch, and a probe current of 100 pA. High-resolution high-angle annular dark field (HAADF) STEM image acquisition was done at the same high voltage and convergence semi-angle conditions.

The in situ electrochemical measurements were conducted using the EC-Lab software installed on a computer with StreamPix software for in situ direct electron imaging to perform synchronized data acquisition. The on-chip Pt reference electrode was calibrated against the bulk Ag/AgCl reference electrode and had an offset of +50 to +200 mV depending on the batch of the wafer. Linear sweep voltammetry was set to run from 0 vs open circuit potential to -0.8 V vs RHE at 20 mV/s scan rate, while chronoamperometry was set to run at -0.8 V vs RHE for 5 min.

The in-situ data post-processing and particle analyses protocol. Imaging with an electron flux rate of 30 $e^{-}nm^{-2}s^{-1}$ allowed to eliminate electron beam induced damage. The resulting low signal to noise ratio images were first time-synchronized with the electrochemical data and then denoised using the noise-to-noise Cryo-CARE method,[3,4] resulting in a temporal resolution of 10 fps. Subsequently, particle analyses were performed using the SAMJ plugin in ImageJ software. The projection area of the primary NCs was measured every 50 slices. The relative area loss was measured for 5 to 8 NCs, then the mean and standard deviation were calculated. For the agglomerated Cu_2O NCs, hand annotations were used for 6 agglomerates every 100 slices and then the same procedure was employed as for the analysis of the other NCs.

Electrochemical Characterization and Electrocatalytic Measurements

Material

Potassium carbonate (K_2CO_3, 99+ %, ACS reagent) was purchased from Acros organics. Potassium hydroxide (KOH, 99.99 trace metal basis) was purchased from Sigma Aldrich. Sodium chloride (NaCl, 99.999% puratrem) was purchased from abcr. Lead (II) nitrate (Pb(NO3)2, 99+%, A.C.S. reagent) were purchased from IVALUA. Nitric acid (HNO3, >= 65%) was obtained from Sigma Aldrich. All electrolytes were prepared with ultra-pure Mili-Q water (resistivity 18 MΩ cm).

All the electrochemical measurements were performed with a Biologic SP-300 Potentiostat. Ag/AgCl electrode (leak-free series, Innovative Instruments) was used as a reference electrode. Before each measurement, the reference electrode was calibrated against Ag/AgCl reference electrode.

Electrode preparation

Glassy carbon plates (2.5 cm × 2.5 cm × 0.3 cm, Sigradur G, HTW) coated with the electrocatalyst were used as the working electrode (cathode), except specified otherwise. Before coating, the glassy carbon plates were polished using an alumina paste (50 nm alumina, BAS Inc.) on a polishing pad. Then, the substrates were rinsed with Milli-Q water and polished again on an alumina-free polishing pad. Following the polishing, the electrodes were cleaned by consecutive sonication (Bandelin Sonorex RK 106, 35 kHz) in Milli-Q water and isopropanol for 10 min each.

The working electrodes (cathodes) were prepared by drop-casting 25µg/cm^2 (here, 32 ug for 1.33 cm^2) of copper-based nanocrystals dispersed in toluene. Nafion was added to Cu_2O NCs to prevent detachments. No carbon support was added to the catalyst. After drop-casting, the electrodes were allowed to dry for 5-10 min. The electrodes were blown dry with N_2 and tested as prepared. The as-prepared working electrode was used for the different electrochemical characterizations, except specified otherwise.

Lead Under Potential Deposition (Pb UPD)

Lead underpotential deposition (Pd UPD) was performed in a custom-made PEEK H-cell with Ag/AgCl electrode (leak-free series, Innovative Instruments) as reference electrode and Pt foil as counter electrode. For each sample, five consecutive cyclic voltammograms were recorded

in 0.1 M KOH with 1 mM $Pb(NO_3)_2$ at 10 $mV.s^{-1}$ following a previously reported protocol.[5,6] In all cases, the NCs film remained intact on the electrode after the measurement.

Potential Electrochemical Impedance Spectroscopy (PEIS)

PEIS was performed to determine the electrochemical cell resistance (R_{cell}) and the charge-transfer resistance (R_{ct}) following our previous protocol.[7] In brief, a 20mV sinus amplitude was applied at open-circuit potential using 10 points per decade from 1MHz to 100Hz. 4 spectra were recorded with 2 spectra from the highest to lowest frequency and 2 spectra from the lowest to highest frequency.

Linear Sweep Voltammetry (LSV)

Linear Sweep Voltammetry was performed before and after every CO_2RR experiment with a scan rate of 10 $mV.s^{-1}$ from 0.2 to -1.9 V vs Ag/AgCl in the same electrolyte as the CO_2RR purged with CO_2. The gas was cut during the LSV acquisition.

Electrochemical Surface Area (ECSA)

Electrochemically active surface area (ECSA) was measured by electrochemical double-layer capacitance (ECDL) before and after every CO_2RR experiment, following our previous protocol.[8] Cyclic voltammograms (CVs) were first recorded between −0.450 and −0.350 V vs Ag/AgCl (in a region where no redox activity takes place) at incremented scan rates between 4 and 32 $mV.s^{-1}$. The geometric current-density values for the charging (J_c) and discharging (J_d) capacitance were taken at −0.40 V vs Ag/AgCl. The difference between these values ($J_{total} = J_d - J_c$ / $\mu A.cm^{-2}$) was plotted against the scan rate, ν ($V.s^{-1}$). The slope of this linear plot yields the capacitance value for the sample, C_{sample} ($\mu F.cm^{-2}$). This process was repeated for a clean glassy carbon electrode in order to obtain a reference capacitance value for a flat surface (C_{GC} = 30 $\mu F.cm^{-2}$). Division of C_{sample} by C_{GC} then gives a surface roughness factor (S.R.F.). The ECSA normalized current density (J_{ECSA} $mA.cm^{-2}$) was calculated by dividing the geometric current density (J_{geo}, $mA.cm^{-2}$) by the S.R.F obtained after CO_2RR.

Chrono Amperometry (CA) for Electrochemical CO_2 Reduction Reaction (CO_2RR)

Electrochemical measurements were performed in a custom-made gas-tight H-cell made of polycarbonate and fitted with Buna-N O-rings. The configuration of the electrochemical cell was such that the working electrode sat parallel with respect to the counter electrode to ensure a uniform potential distribution across the surface. A Selemion AMV anion exchange

membrane was used to separate the anodic and cathodic compartments. Each of the compartments in this cell contained a small volume of electrolyte (2 mL each) to concentrate liquid products. One chronoamperometry at the desired potential was performed with a constant CO_2 flow rate of 5 sccm. PEIS, LSV and ECDL were performed before and after each chronoamperometry. Dynamic iR-compensation at 85% was consequently applied to all experiments using the cell resistance from PEIS.

Products quantification:

Gas products were separated and analyzed with the use of a gas chromatograph (GC, SRI Instruments) equipped with a HayeSep D porous polymer column, thermal conductivity and flame ionization detectors. The exhaust of the catholyte compartment is directly connected to the inlet of the GC. The GC was calibrated for H_2, CO, CH_4, C_2H_4, and C_2H_6. Five standard gas mixtures (Carbagas) were used to obtain the calibration plots for gaseous product concentration determination. Ultra-high purity N_2 (99.999%) was used as a carrier gas.

Liquid products were isolated by sampling the electrolyte post-electrolysis in both catholyte and anolyte compartments. The necessity to collect electrolyte from both compartments is reasoned by the possible product crossover from the catholyte to the anolyte (i.e., formic acid and acetic acid, which exist in their anionic forms, namely, formate and acetate, in the neutral solution and can migrate through the anion exchange membrane, which separates the two compartments). Liquid products were separated and quantified with the use of high-performance liquid chromatography (HPLC) and a refractive index detector. An Aminex HPX-87H (BioRad) column and a 5 mM H_2SO_4 eluent were used to separate the carboxylic acid and alcohol products. A calibration curve to quantify ethanol, n-propanol, ethylene glycol, formic acid and acetic acid was established by mixing a series of calibration standards in a 0.1 M $KHCO_3$ electrolyte.

In-situ surface-enhanced Raman spectroscopy (SERS)

In-situ surface-enhanced Raman spectroscopy (SERS) was performed with a longue working distance objective (x50L) using a Renishaw inVia Raman microscope. The spectra were collected using a 785 nm laser. To avoid damaging the samples, the spectra were collected a range centered at 1700cm^{-1} using 0.5% full laser intensity with 2% defocusing, 0.1 s integration time, and 500 repetitions. The focus was re-adjusted manually every potential. A different region on the electrode was used for each potential to minimize damaging the sample. The raw data were base corrected manually by using Origin 2019 software using a linear baseline between the two end points of each spectrum. An open-structured H-cell (see schematic below) was utilized for the measurements. CO_2 was bubble at a flow rate of 5sccm and 0.1M $KHCO_3$, CO_2 saturated was used as electrolyte. An Ag/AgCl electrode (filled with saturated aqueous KCl solution) and a platinum wire were used as the reference and counter electrode, respectively.

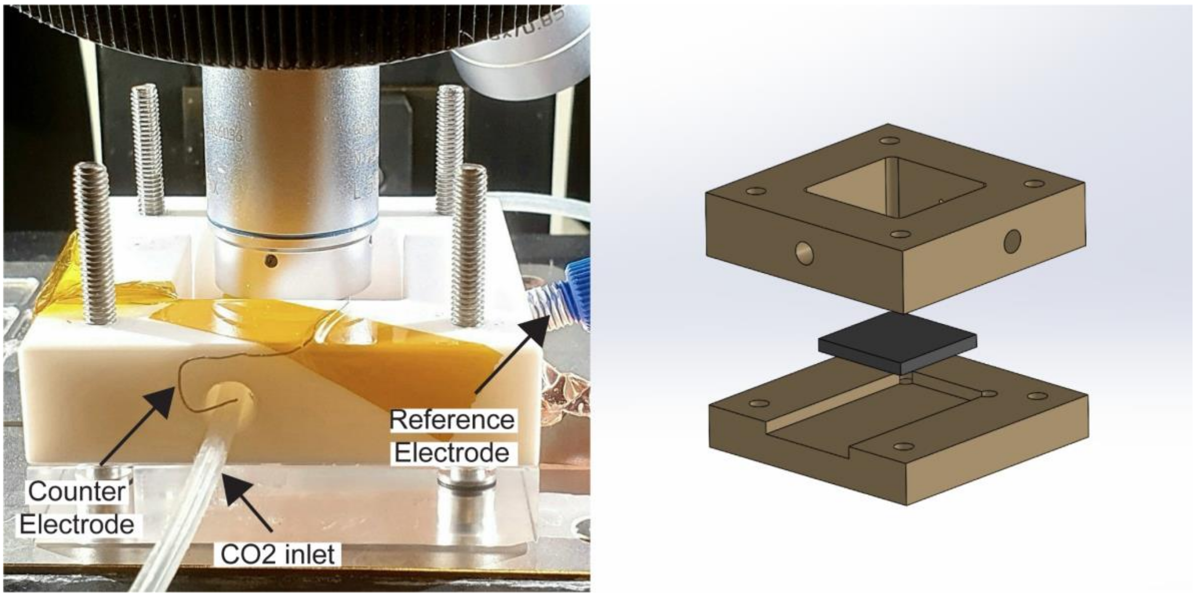

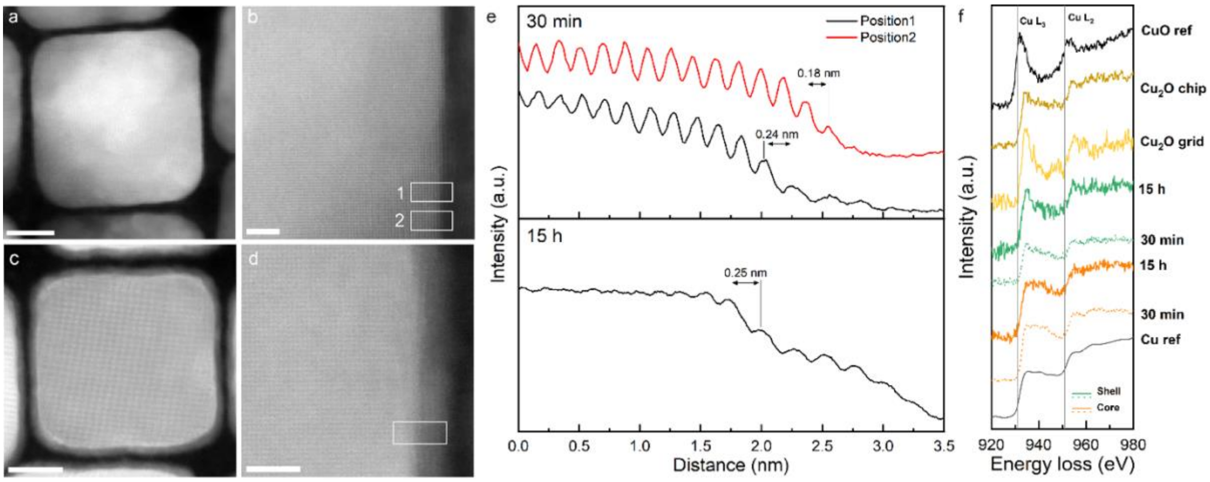

Figure S1. STEM EELS analysis of Cu NCs at different time of OCP exposure. a. HAADF STEM image of Cu NCs after 30 min at OCP in CO_2-saturated 0.1 M $KHCO_3$ electrolyte. **b.** High-resolution HAADF STEM image of the same Cu NCs in (**a**). Scale bar, 2 nm. **c.** HR HAADF STEM image of Cu NCs after 15 h at OCP in CO_2-saturated 0.1 M $KHCO_3$ electrolyte. **d.** Zoomed-in edge of Cu NCs in (**c**). Scale bar, 2 nm. **e.** Line profile of insets in (**b**) and (**d**) showing d-spacings of Cu and Cu_2O. **f.** EEL spectra of Cu $L_{3,2}$ edge of 30 min and 15 h OCP oxidized Cu NCs probed in the shell and core of NCs shown along with reference spectra of Cu, Cu_2O taken on chip and grid, and CuO.

These data show that the thickness increase of the patchy oxide shell, which reaches a steady-state of around 1.5 nm after 15 h at OCP.

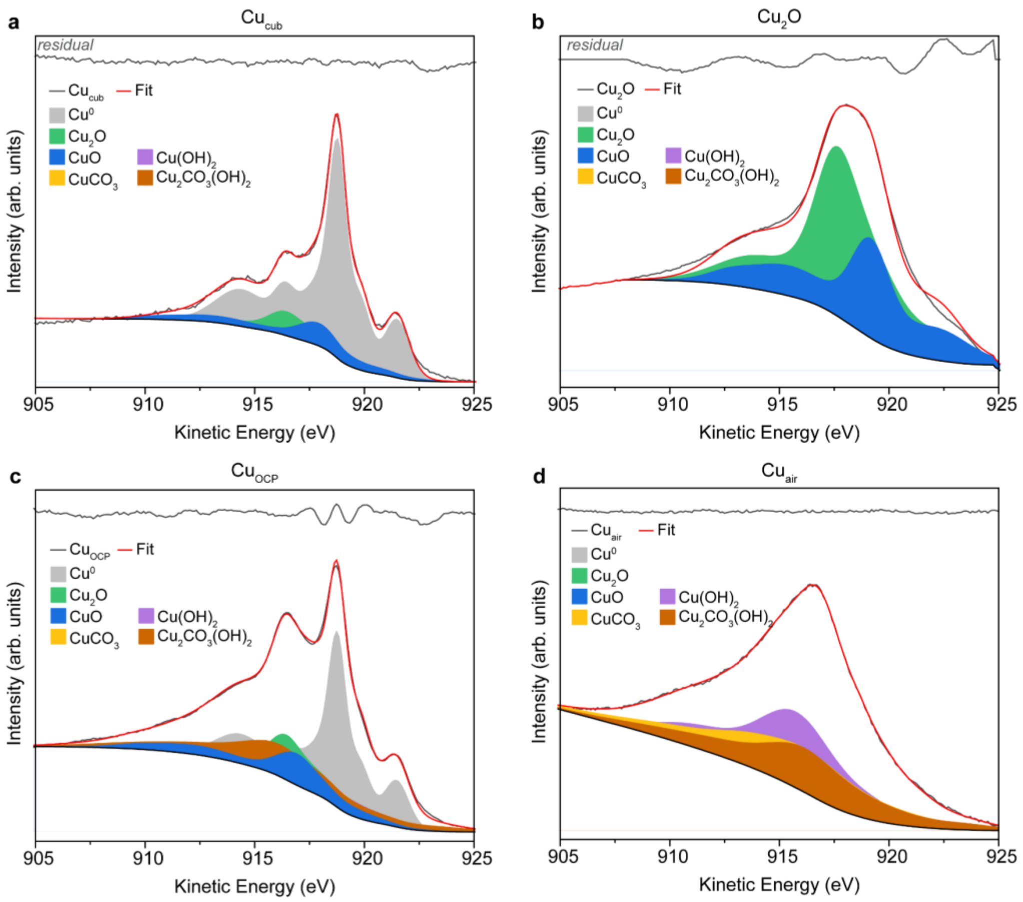

Figure S2. Cu LMM X-ray Auger electron spectroscopy (XAES) a. Cu_{cub} NCs. **b.** Cu_2O NCs. **c.** Cu_{OCP} NCs and **d.** Cu_{air} NCs. The Cu LMM was fitted following the established procedure with the reported standards from previous work, except $CuCO_3$ and Cu_2CO_3OH which were acquired from commercial standard.[9]

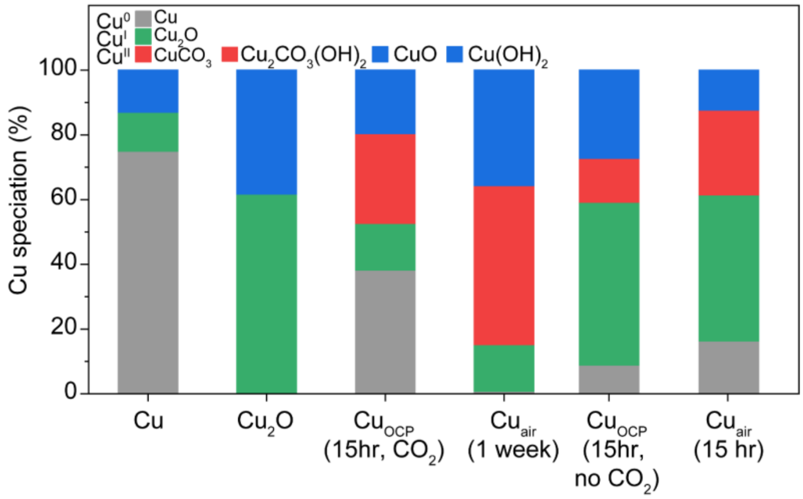

Figure S3. Cu surface speciation from LMM X-ray Auger electron spectroscopy (XAES).

The Cu surface speciation from LMM X-ray Auger electron spectroscopy (XAES) revealed that the fraction of Cu-carbonate increases with CO_2 purging at OCP (Cu_{OCP}, 15 hours, CO_2 vs no CO_2) and with time during air exposure (Cu_{air}, 15 hours vs 1 week).

Table S1. Faradaic Efficiency (%) of Cu NCs, Cu_2O NCs, Cu_{OCP} NCs and Cu_{air} NCs at -1.1 V vs RHE for 1 and 10 h chronoamperometry in H-cell in 0.1 M $KHCO_3$ with corresponding standard deviation calculated from three independent measurements.

1-hour chronoamperometry

Sample	H_2	std.	CO	std.	CH_4	std.	C_2H_4	std.
**Cu NCs**	34.26	5.5	2.32	0.78	11.86	2.92	28.26	4.04
**Cu_2O NCs**	33.61	5.78	2.39	0.93	11.01	2.26	31.19	2.35
**Cu_{OCP} NCs**	30.18	4.3	2.88	0.31	16.16	6.29	21.83	1.33
**Cu_{air} NCs**	67.63		4		5.7		15.93	

$HCOO^-$	std.	Acetate	std.	Ethylene Glycol	std.	EtOH	std.	1-PrOH	std.
8.58	3.24	0.31	0.53	0	0	4.46	3.95	3.36	1.27
6.58	1.1	0.78	0.7	0.04	0.07	7.68	0.8	4.23	0.73
11.18						4.27		1.75	

10 hours chronoamperometry

Sample	H_2	std.	CO	std.	CH_4	std.	C_2H_4	std.
**Cu NCs**	31.4	3.48	3.59	0.93	7.68	1.26	26.15	5.47
**Cu_2O NCs**	34.49	1.73	2.02	0.71	7.79	3.19	11.45	0.37
**Cu_{OCP} NCs**	46.41	9.63	3.34	2.06	5.83	1.63	4.87	3.51
**Cu_{air} NCs**	62.08		4.28		5.44		8.33	

$HCOO^-$	std.	Acetate	std.	Ethylene Glycol	std.	EtOH	std.	1-PrOH	std.
5.55	1.52	0.68	0.13	0.15	0.02	5.83	2.28	3.17	0.53
8.22	0.46	0.44	0.03	0.18	0.06	3.49	1.59	1.62	0.75
10.49	1.71	0.06	0.08	0.14	0	1.85	1.08	0.71	0.32
3.77		0.3		0.04		0.56		0.78	

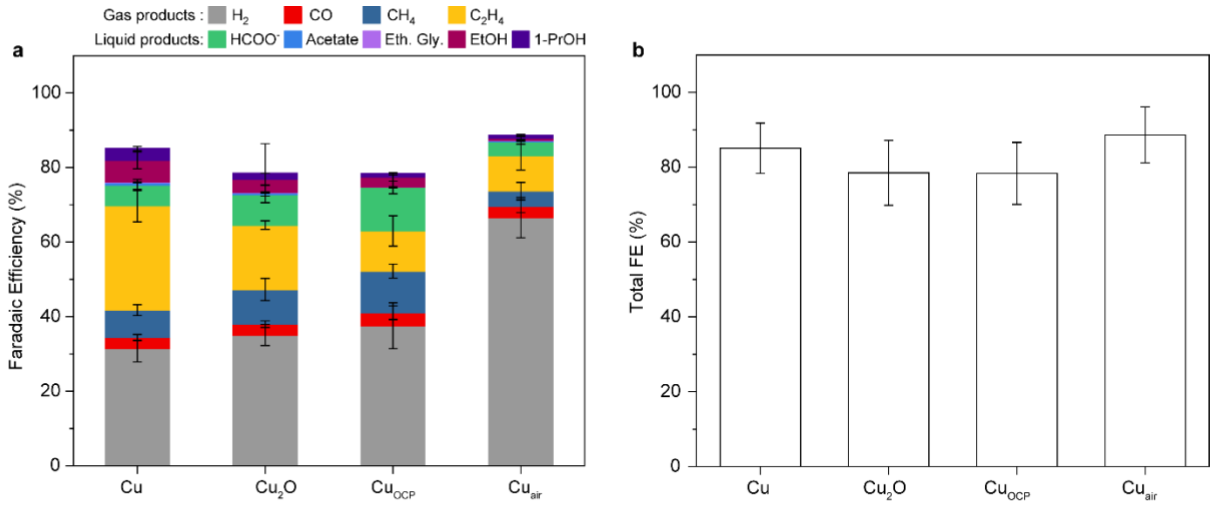

Figure S4. a. FEs for all gaseous products (that is, H_2, CO, CH_4, and C_2H_4) **and the main liquid products** (that is, $HCOO^-$, Acetate, Ethylene glycol, C_2H_5OH, and 1-propanol) at -1.1 V vs RHE average over 10 hours of chronoamperometry measurements for the different catalysts **b. Total FE average over 10 hours.** The data are the average of three independent experiments, and the error bars are the calculated standard deviation. The total FE efficiency is lower than 100% for all samples because of migration of ionic products (i.e. formate) and possibly because of liquid products being averaged over 10 hours while gases are averaged over the last hour in (a).

When compared to the selectivity data after 1 hour and as highlighted by the product trends reported in Figure 2, the selectivity of the Cu NCs remains rather stable. The changes in the product distribution of the Cu_2O NCs are more visible with a marked decline in ethylene FE, which drops to 11%. The change with time of ethylene FE for both metallic Cu cubes and Cu_2O agrees well with previously published studies.[10–12] Even more drastically, the FE of CO_2RR gas products for Cu_{OCP} is nearly fully suppressed and the competing hydrogen evolution reaction (HER) dominates after 10 hours. The performance of Cu_{air} NCs do not change substantially and HER dominates the entire time.

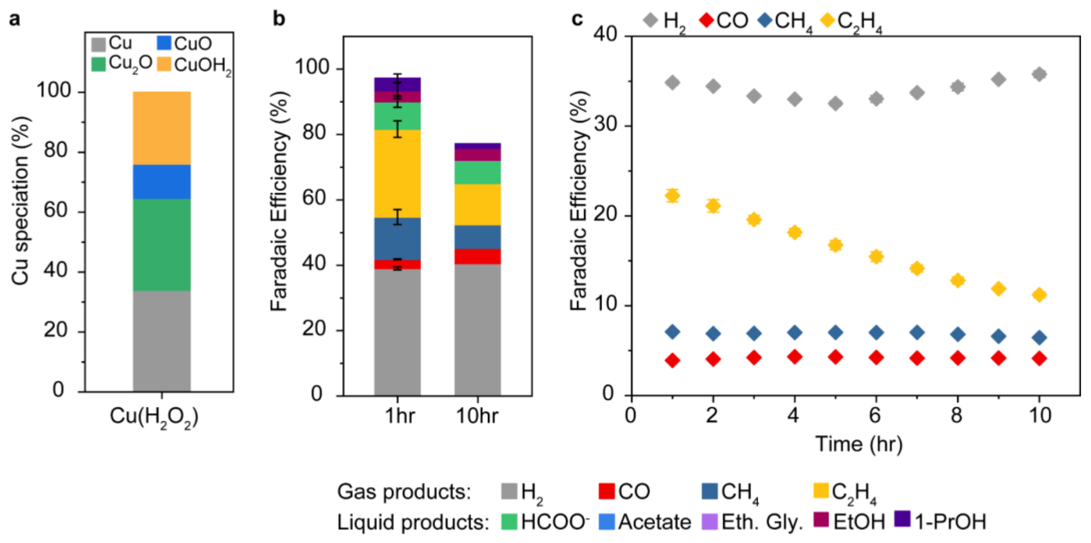

Figure S5. Surface composition and CO_2RR performances of surface oxidized Cu NCs. a. Surface speciation from Cu LMM XAES**. b.** Total FEs for all gaseous products (that is, H_2, CO, CH_4 and C_2H_4) and the main liquid products (that is, $HCOO^-$, Acetate, Ethylene glycol, C_2H_5OH and 1-propanol) at -1.1 V vs RHE for 1 and 10 h. **c.** Evolution of the FEs for all gaseous products (that is, H_2, CO, CH_4 and C_2H_4) during 10 h of CO_2RR.

Oxidation with H_2O_2 was performed on the Cu NCs to generates similar surface composition as the Cu_2O NCs. This surface oxidation was performed following our previous work.[8] In brief, a suspension of 3 mg.L^{-1} of as-synthesized Cu NCs (typically 30 mg in 10 mL Toluene) was oxidized by introducing a dilute solution of hydrogen peroxide in ethanol (in 1:1 ratio H_2O_2 to Cu atoms on the surface). The mixture was allowed to react for 5 to 10 min before purifying the reaction mixture by centrifugation at 6000 rpm for 8 min. The particles were re-dispersed using around 2 mL of toluene and were stored in an N_2-filled glovebox.

The data in **Figure S5** evidence that the surface modification of the Cu NCs to display OD active sites results in a selectivity and operational stability similar to Cu_2O NCs as they undergo faster selectivity loses compared to the original metallic Cu NCs.

Supplementary Note 1:

Several studies report higher selectivity toward C_{2+} products (i.e., ethylene and ethanol) during CO_2RR for oxide-derived copper compared to metallic copper.[13–18]

However, we note that the starting copper oxide and the metallic copper electrodes often differ in terms of morphology/exposed facets, roughness/particle size, and loading.[16,19–22] These differences make the comparison not always trivial.

Actually, when comparable electrodes are used, the improvement in C_{2+} selectivity is not always observed.[23,24] For example, Cu foil and oxidized Cu foil displayed little to no change in C_{2+} selectivity.[23] Similarly, no improvement in C_{2+} selectivity was observed among Cu and O(I)D-Cu and O(II)D-Cu obtained from anodically polarizing the same Cu electrode.[24]

The enhanced C_{2+} selectivity mentioned above, along with sometimes increased intrinsic activity, has been generally attributed to the increase in roughness and generation of defects and grain boundaries, triggered by the oxygen removal from the Cu lattice upon reduction, favoring C_{2+} products.[13–16,20,25] When the metallic Cu electrode is already nanostructured/defective, the selectivity improvement achieved from the oxide-derived electrode has been observed to be lower.[11,19,20,26]

Our Cu, Cu_2O (**Figure 2**), and oxidized Cu cubes (**Figure S5**) display similar initial selectivity toward the C_{2+} products. The selectivity is consistent with what was reported in previous literature.[1,6,11,12,26] The observation of similar C_{2+} selectivity among oxide-derived and metal-derived nanostructured electrodes is in line with previous literature.[17,19,23,24]

Then, clear differences emerge in the operational stability over 10 hours, wherein the oxidized Cu cubes (**Figure S5c**) and the Cu_2O cubes (**Figure 2c**) deactivate faster compared to the metallic Cu cubes (**Figure 2b**). The effect of the oxide on operation stability has been less investigated, so comparison with the literature is not possible, to the best of our knowledge.

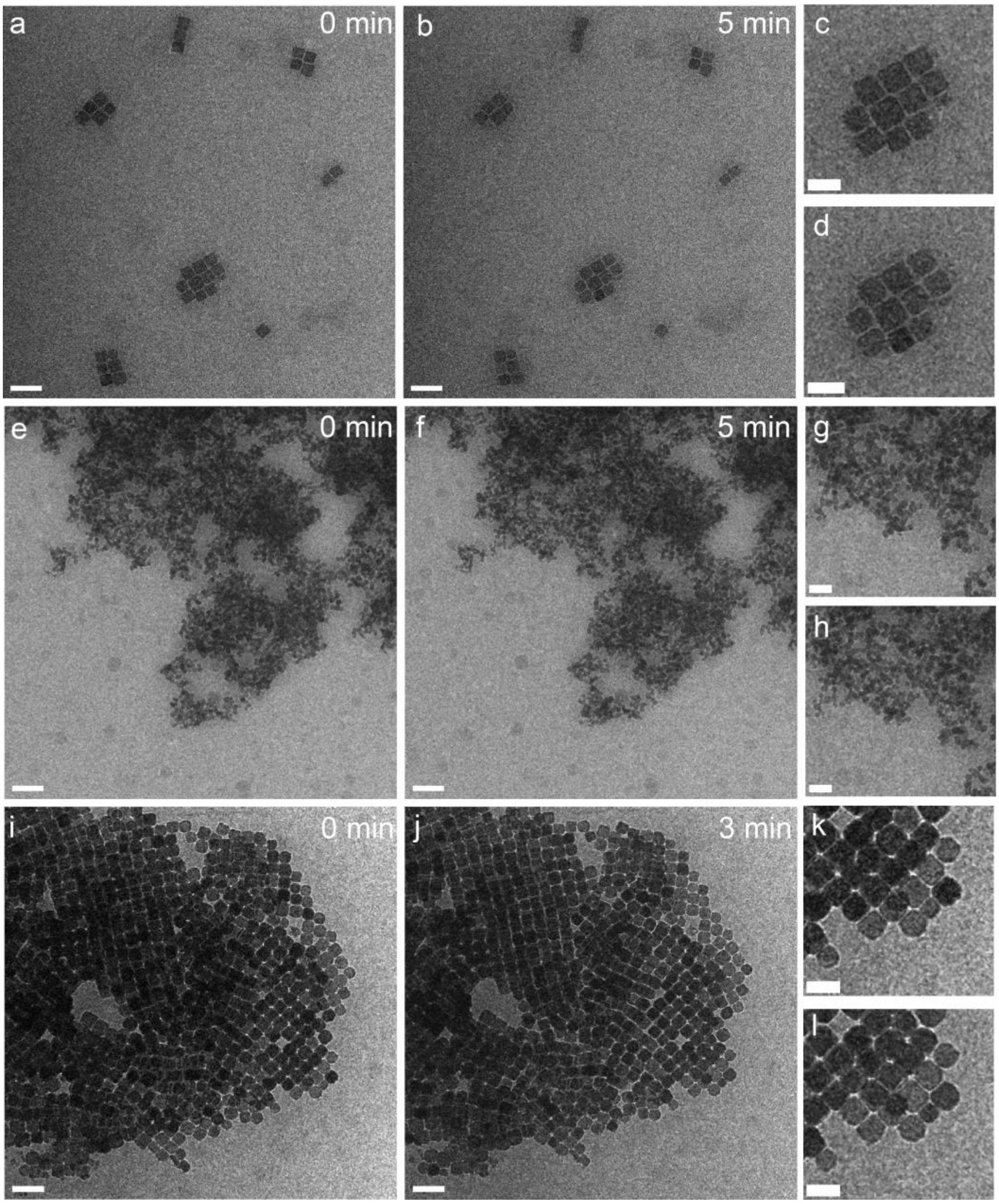

Figure S6. ec-LPTEM control experiments without applied potential. a,b Cu NCs before (**a**) and after (**b**) continuous imaging at 30 $e^{-}nm^{-2}s^{-1}$. Scale bar, 100 nm. **c,d** are zoom-in of (**a**) and (**b**), respectively. Scale bar, 50 nm. **e,f** Cu_2O NCs before (**e**) and after (**f**) continuous imaging at 25 $e^{-}nm^{-2}s^{-1}$. Scale bar, 200 nm. **g,h** are zoom-in of (**e**) and (**f**), respectively. Scale bar, 100 nm. Cu_{air} NCs before (**i**) and after (**j**) continuous imaging at 30 $e^{-}nm^{-2}s^{-1}$. Scale bar, 100 nm. **k,l** are zoom-in of (**i**) and (**j**), respectively. Scale bar, 50 nm.

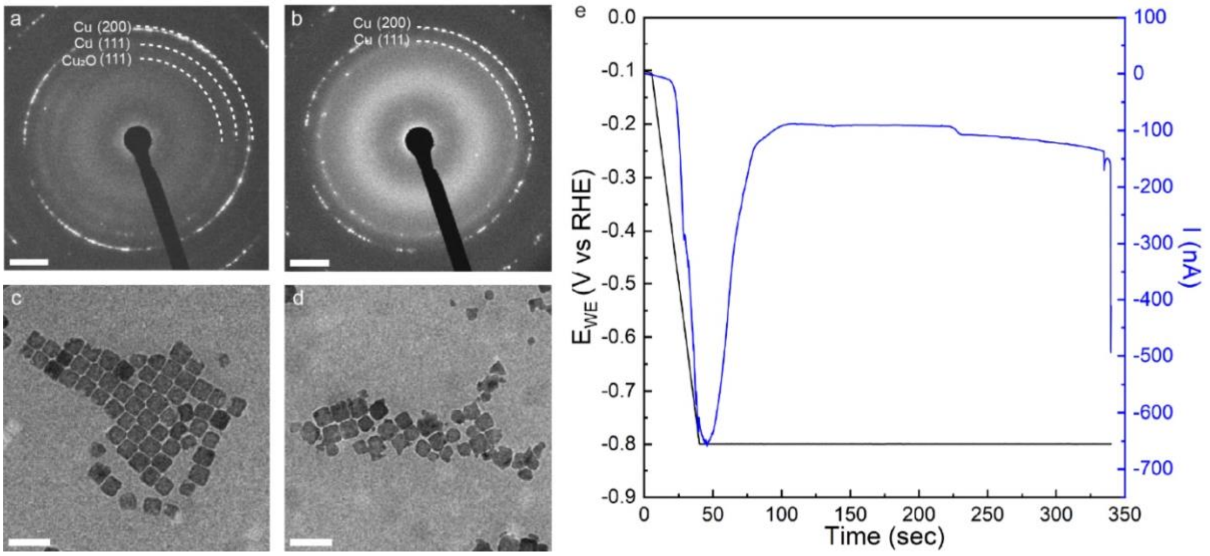

Figure S7. TEM characterization of Cu NCs before and after ec-LPTEM during CO_2RR. **a, b.** Energy-filtered SAED of Cu NCs (**a**) before and (**b**) after LSV-CA measurement. Scale bar, 2 nm^{-1}. **c,d.** Respective images of Cu NCs before (**c**) and after (**d**) LSV-CA measurement. Scale bar, 100 nm. **e.** Potential and current profile recorded during LSV and CA measurements at -0.8 V vs RHE of Cu NCs.

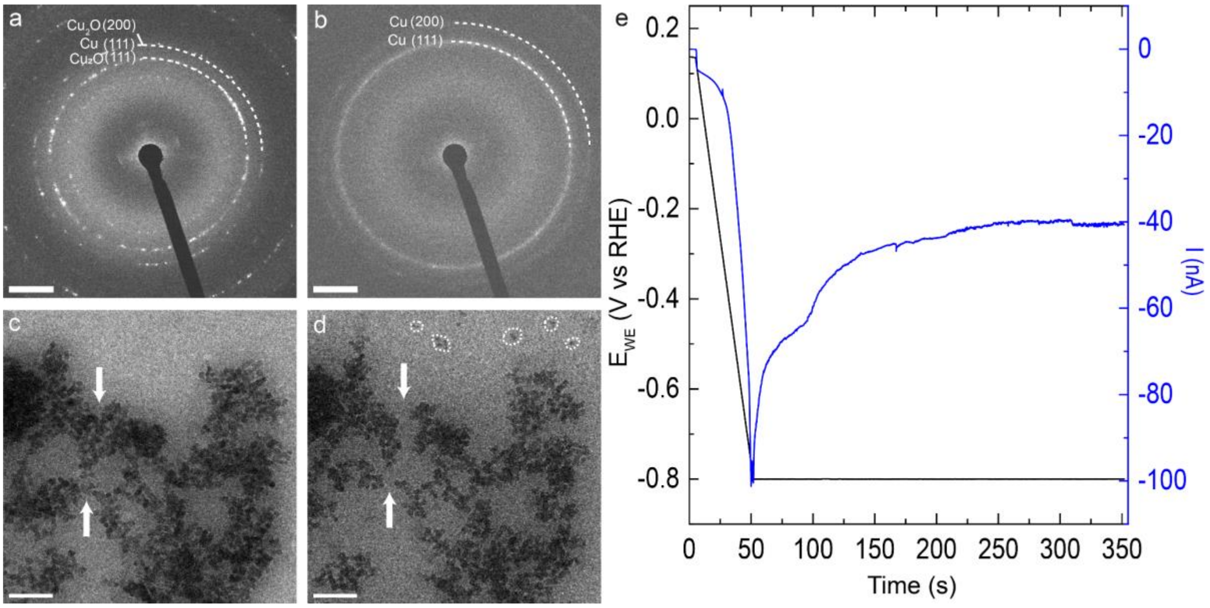

Figure S8. TEM characterization of Cu_2O NCs before and after ec-LPTEM during CO_2RR. a,b. Energy-filtered SAED of Cu_2O NCs (**a**) before and (**b**) after LSV-CA measurement. Scale bar: 2 nm^{-1}. **c,d.** Respective images of Cu_2O NCs before (**c**) and after (**d**) LSV-CA measurement. White arrows indicate on regions, where particles agglomerates get separated due to dissolution/shrinkage. Scale bar, 100 nm. **e.** Potential and current profile recorded during LSV and CA measurements at -0.8 V vs RHE of Cu_2O NCs.

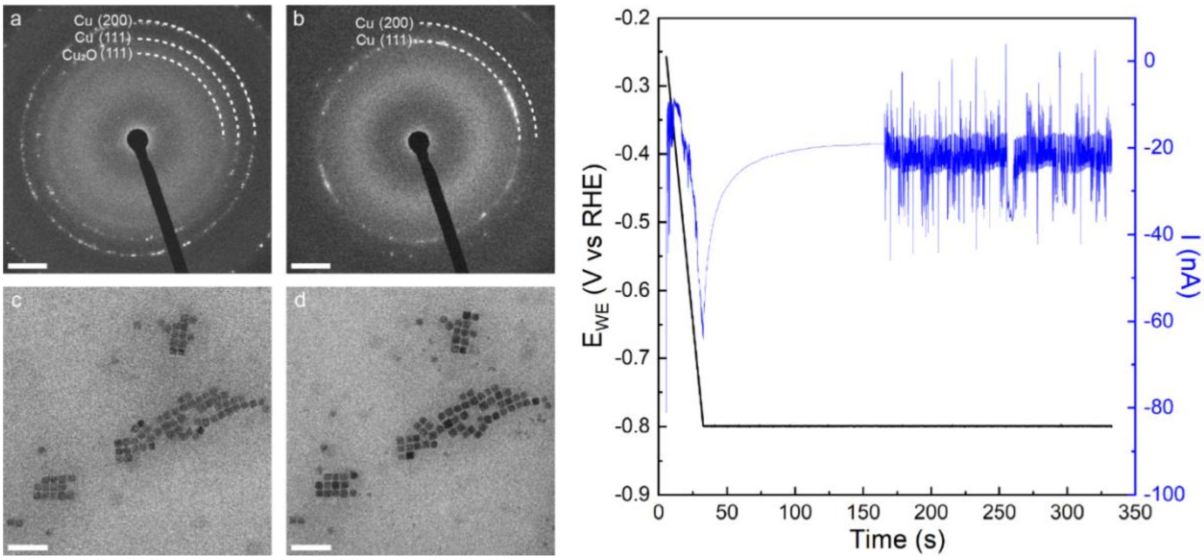

Figure S9. TEM characterization of Cu_{OCP} NCs before and after ec-LPTEM during CO_2RR. (**a,b**) Energy-filtered SAED of Cu NCs (a) before and (b) after LSV-CA measurement. Scale bar, 2 nm-1. (c) and (d) Respective images of Cu NCs before and after LSV-CA measurement. Scale bar, 200 nm. (e) Potential and current profile recorded during LSV and CA measurements at -0.8 V vs RHE.

Zirconium oxide coating

In this work, we find that the presence OD active sites on the Cu NCs accelerates the operational instability. Previous work evidence that coatings of AlOx or ZrO_x synthesized via colloidal atomic layer deposition (c-ALD) render Cu NCs structurally more stable during CO_2RR by locking a fraction of surface oxide at operating conditions.[6,8,27] Here, we utilized Cu@ZrOx as a comparison sample to demonstrate its stability during operation using ec-LPTEM. The data are summarized in **Figure S10-S12**.

The Cu@ZrOx NCs were synthesized following our previous work.[6,8,27] In brief, 800µg of pre-oxidized Cu(H_2O_2) NCs were diluted in 9 ml of toluene and stirred under N_2 flow. Two solutions of ZrO_x precursor and H_2O/OLAC diluted in octane and dioxane respectively were charged into separated, dried, N_2-flushed gas-tight syringes. The ZrO_x concentration was 80µmol.L^{-1} (for the first 1.8 mL) to 0.4mmol.L^{-1} (for the following 2mL). The H_2O/OLAC solution was made as such that [H2O]/[TMA] = 2:3 and [OLAC]/[Zr] = 1:3. Both solutions were injected in parallel with a syringe pump at 1mL.h^{-1} A mass ratio Cu: Zr of around 2-3% (corresponding to a 1:1 ratio Cu on the surface to Zr) was targeted to ensure uniform encapsulation of the NCs.

First, the data presented in **Figure S10** and **S11** evidence the presence of an amorphous and porous ZrO_x shell surrounding the Cu NCs, in line with our previous studies.[6,8,27]

Second, the data in **Figure S12** show that the projected area and the average size before and after LSV-CA of the Cu@ZrO_x NCs remain the same during the start-up and operational phases as opposed to the Cu and Cu_2O NCs (**Figure 3 and Figure S6-9**). Additionally, no redeposition of secondary particle are observed in the surrounding. The quasi-operando monitoring of the Cu@ZrO_x NCs confirm that the oxide shell act as a mask that mitigate the structural reconstruction during CO_2RR, most likely by confining within the vicinity of the surface the transient species responsible for the dissolution-redeposition cycles. Similar conclusions were highlighting the role of thin encapsulating oxide overlayers as a physical barrier to mass transport do volatile Pt atoms causing Ostwald ripening during thermal oxidation reaction.[28,29]

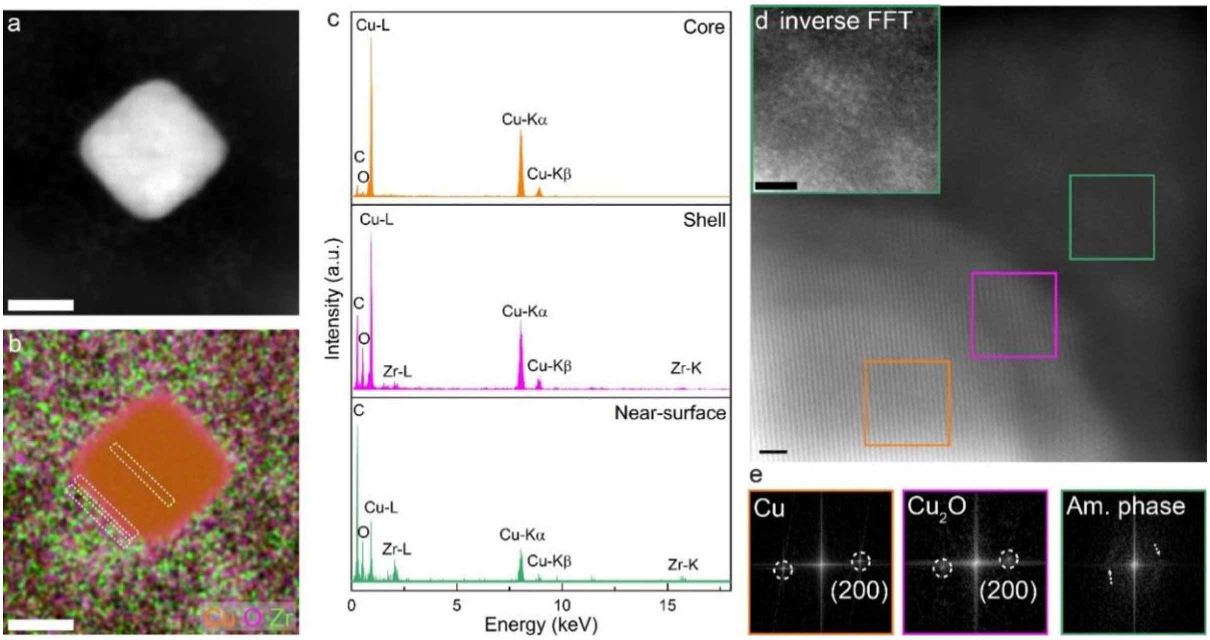

Figure S10. Characterization of Cu@ZrOx NCs catalyst. a. HAADF STEM image of Cu@ZrOx NCs. Scale bar, 20 nm. **b.** Corresponding overlapped EDS map of Cu, O, and Zr. Scale bar, 20 nm. **c.** EDS spectra from NCs core, shell, and near-surface area regions outlined in (**b**). **d.** High-resolution STEM image of Cu@ZrOx NCs. Scale bar, 2 nm. **e.** FFT from regions outlined with orange, magenta, and green squares corresponding to Cu core, Cu_2O shell, and amorphous ZrO_x region, respectively. Inset in (**d**) represents inverse FFT from the amorphous region. Scale bar, 1 nm.

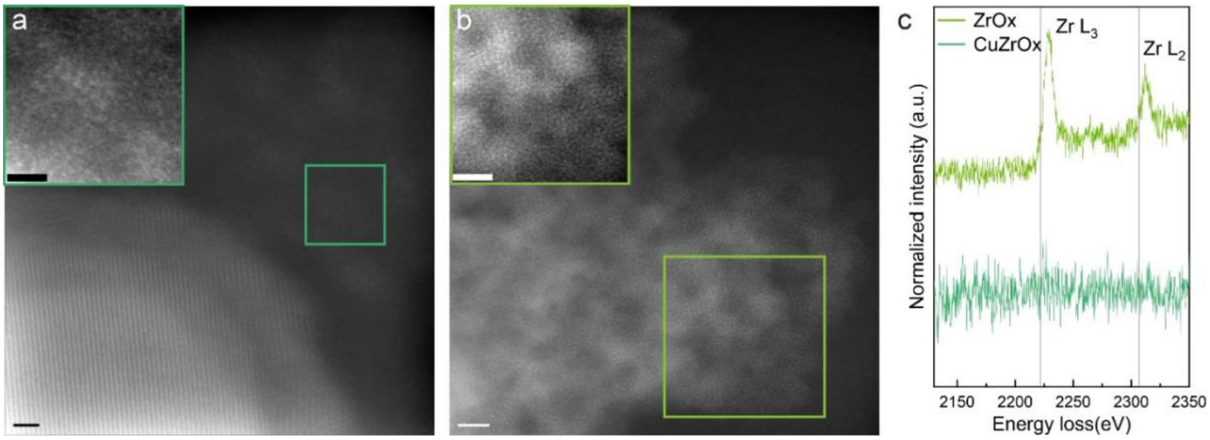

Figure S11. EELS analysis of Cu@ZrOx NCs and bulk ZrO_x. a. HR STEM image of Cu@ZrO_x NCs. Scale bar, 2 nm. Inset represents the inverse FFT from the amorphous region outlined with a green square. Scale bar, 1 nm. **b.** HR STEM image of ZrO_x. Scale bar, 2 nm. Inset represents the inverse the FFT from the region outlined with light green square. Scale bar, 1 nm. **c.** EELS spectra of Zr $L_{3,2}$ edge of $CuZrO_x$ and ZrO_x samples.

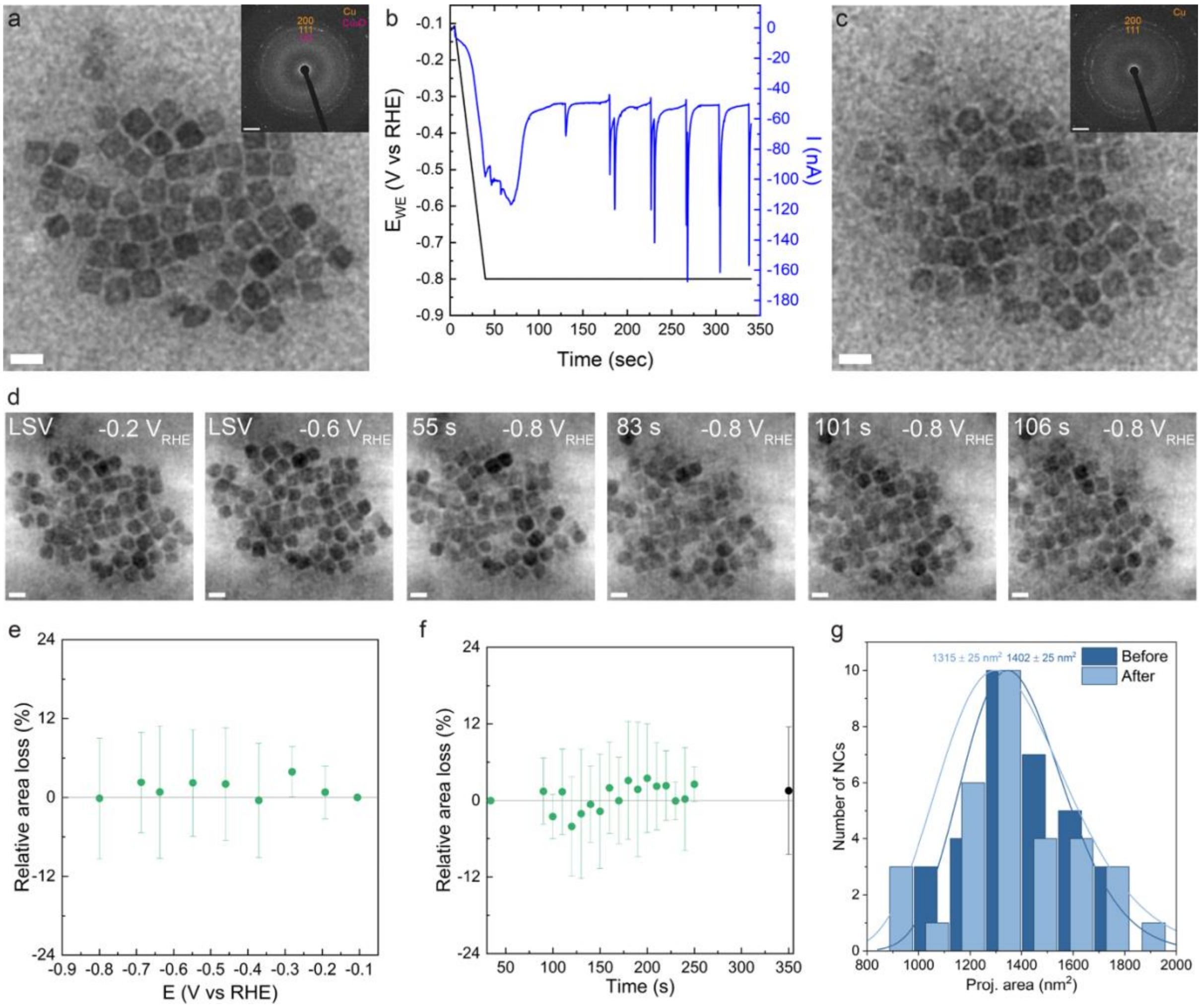

Figure S12. ec-LPTEM experiments on Cu@ZrO$_x$ NCs at CO$_2$RR relevant conditions. a. In situ TEM image of Cu@ZrO$_x$ NCs at OCP. Scale bar, 50 nm. The inset represents the corresponding selected area electron diffraction. Scale bar, 2 nm^{-1}. **b.** Current and potential profiles during LSV and CA measurement. **c.** In situ TEM image of Cu@ZrO$_x$ NCs after LSV-CA measurement. Scale bar, 50 nm. The inset represents the corresponding selected area electron diffraction. Scale bar, 2 nm^{-1}. **d.** Time-lapse image series of Cu@ZrO$_x$ NCs synchronized with LSV-CA measurement. Scale bar, 50 nm. **e,f.** Represent potential- and time-resolved relative area loss of Cu@ZrO$_x$ NCs during LSV (**e**) and CA (**f**) measurements, respectively. The black data point in (**f**) corresponds to the relative area loss of particles measured after LSV-CA measurement. (**g**) Histograms depicting the distribution of projection area of Cu@ZrO$_x$ NCs before and after LSV-CA measurement.

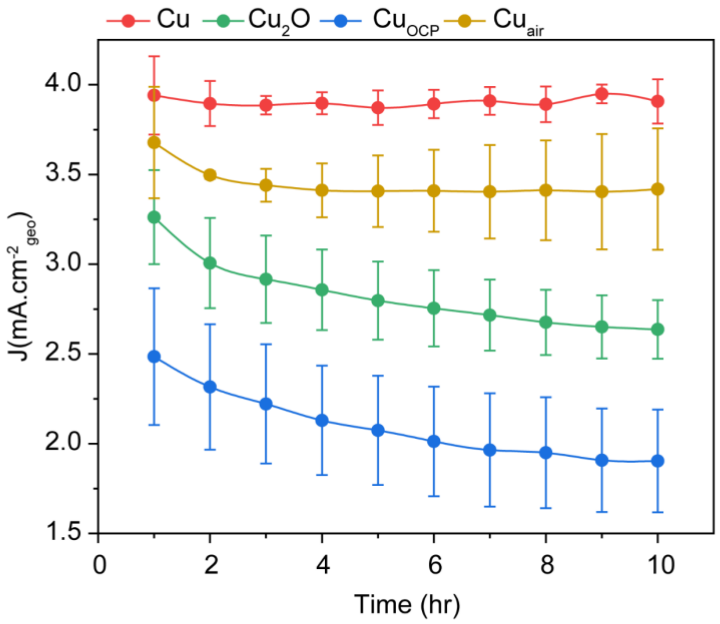

Figure S13. Total current density over 10 h of CO_2RR. We observed a decrease in the total current density which is associated with a decrease in catalytic activity as we observed no physical detachment of the catalysts.

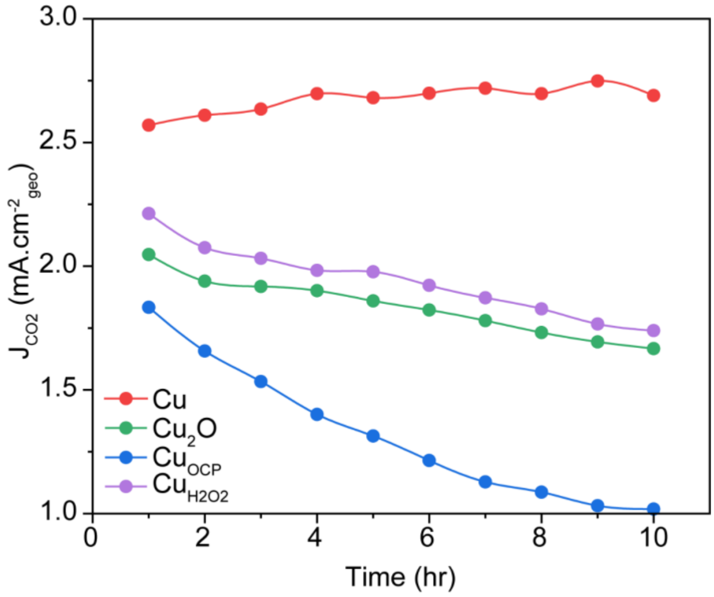

Figure S14. Evolution of the CO_2 partial current density normalized by the geometric area as a function of time.

We report the evolution of the $J_{CO2\text{-}geo}$ over time as a metric to discriminate catalyst deactivation rates and added Cu_{H2O2} in **Figure S14.**

Cu_{H2O2} are surface oxidized Cu cubes with surface composition including oxide but no carbonate. The metallic content is similar to Cu_{OCP} from XAES (**Figure S5**). The observation that Cu_{H2O2} deactivates similarly to Cu_2O agrees with the conclusion that carbonates play a key role in deactivation. If oxides were more important, Cu_{H2O2} would deactivate similarly to Cu_{OCP} and Cu_2O would be expected to be the fastest to deactivates, which is not the case.

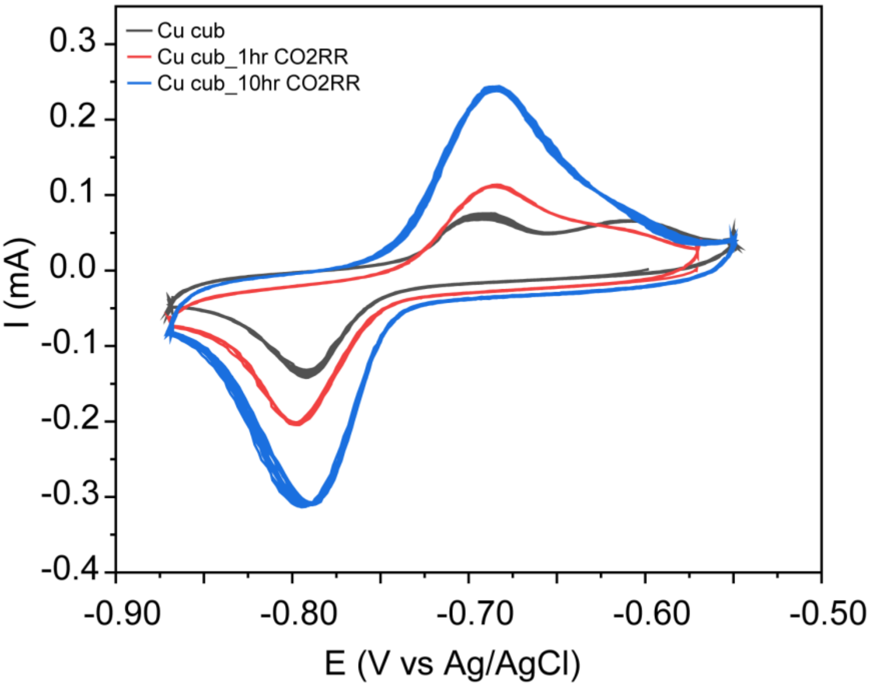

Figure S15. Representative Pb underpotential deposition before and after 1 and 10 h CO_2RR reported for the Cu NCs.

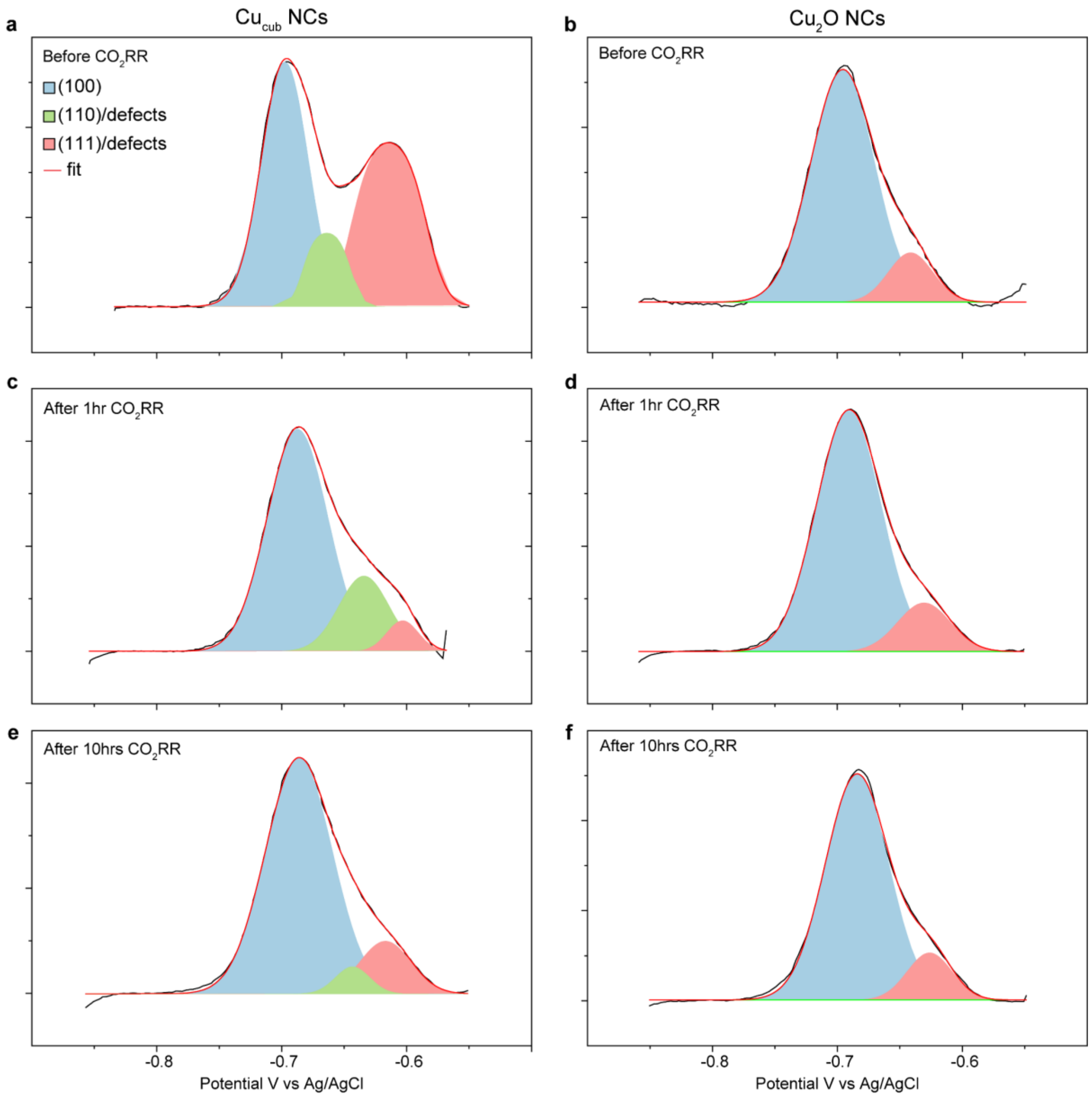

Figure S16. Facets distribution from Pb-UPD. a,b. Pb UPD of Cu$_{cub}$ NCs before CO_2RR (**a**) and of Cu_2O NCs after 5min CO_2RR to reduce the Cu surface (**b**). **c,d.** Pb-UPD after 1hr of CO_2RR for Cu$_{cub}$ (**c**) and Cu_2O (**d**) NCs. **e,f.** Pb-UPD after 10hrs of CO_2RR for Cu$_{cub}$ (**e**) and Cu_2O (**f**) NCs.

The facet distributions of Cu$_{cub}$ and Cu_2O NCs were retrieved by fitting the oxidative peak from Pb-UPD with a Gaussian.[30] A linear background subtraction was used. We attributed the 3 distinct peaks to (100), (110), and (111). Yet, we stress that the peaks for (110) and (111) do not correlate too facet explicitly but rather to defects from edges, corners, and undercoordinated sites. The results are summarized in **Figure S17**.

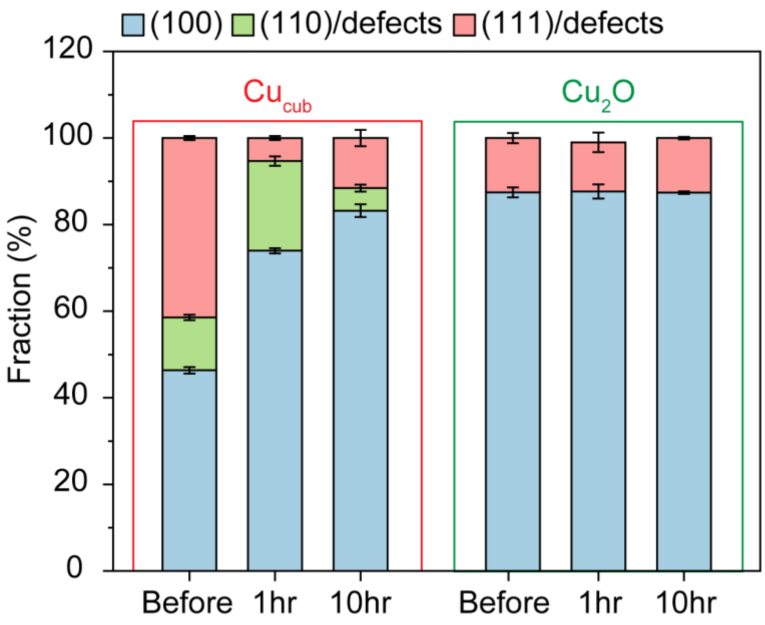

Figure S17. Facet distribution for Pb-UPD for Cu and Cu_2O NCs before and after 1 and 10 h of CO_2RR.

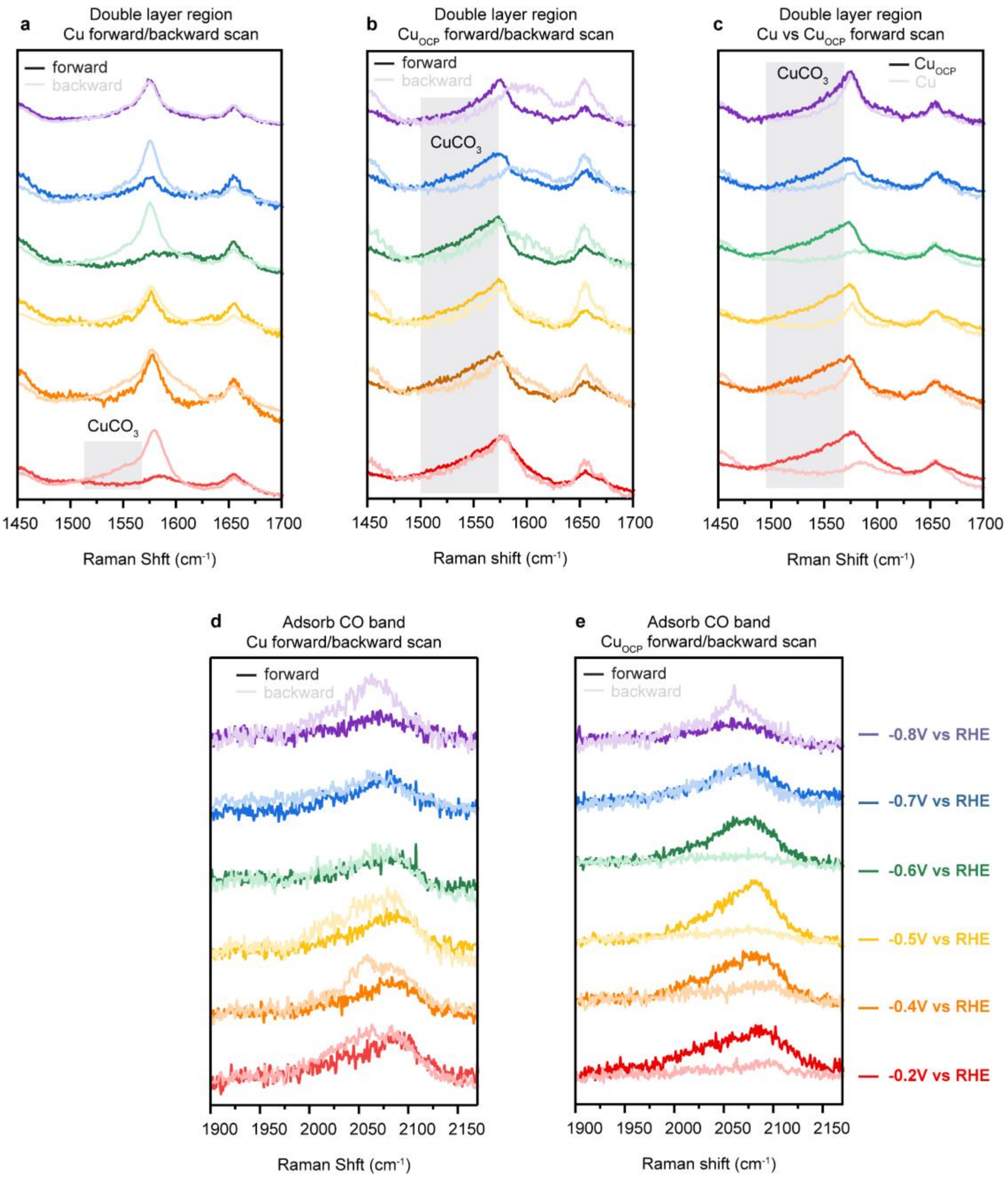

Figure S18. In-situ surface-enhanced Raman spectroscopy (SERS) of Cu and Cu$_{OCP}$ recorded during stepwise decrease of the potential from -0.2V to -0.8V vs RHE and subsequent increase back to -0.2 V vs RHE. **a,b.** Forward and backward scan in the double region scan for Cu (**a**) and Cu$_{OCP}$ (**b**) NCs. **c**. Overlay of the double-layer range of Cu and Cu$_{OCP}$ during forward scan. **d,e.** Forward and backward scan of the CO band for Cu (**d**) and Cu$_{OCP}$ (**e**) NCs.

One recent study has shown that Cu-carbonate species exhibit more cathodic reduction potentials than Cu-oxide counterparts, suggesting that Cu-carbonate may persist under operating conditions.[31]

We monitored the behavior of the carbonate species by in situ surface-enhanced Raman spectroscopy with the sole goal of supporting the idea that copper-carbonate persist under operating potentials, thus impacting the behavior of the investigated copper catalysts (SERS, **Figure S18** and **Table S2**). We chose Cu NCs, as a reference with no surface carbonate, and Cu_{OCP}, as the sample with higher carbonate fraction, yet still CO_2RR active, based on the XAES data (**Figure 1**).

We applied a potential ramp from -0.2 to -0.8 V vs RHE in both forward and backward, with each potential held for 10 min, which are similar operation conditions to those employed in the quasi-operando liquid phase TEM. Given our goal, we focused primarily on the double-layer range where carbonate-related bands appear (**Figure S18a-c**). We measured metallic Cu cubes (**Figure S18a**) and Cu oxidized at OCP (Cu_{OCP}, **Figure S18b**). We compared them by overlaying the forward scan of both samples (**Figure S18c**).

Both samples exhibit bands at 1575 cm^{-1} and 1660 cm^{-1}, previously assigned to adsorbed carboxylates and water bands.[2,32–36] However, Cu_{OCP} exhibits a much more pronounced shoulder at lower wavenumbers, near 1540 cm^{-1}. This shoulder persists until -0.7 V vs RHE and remains evident during the backward scan starting from -0.6 V vs RHE (**Figure S18b**).

This shoulder at lower wavenumbers than the carboxylate near 1540 cm^{-1} has been attributed to Cu-carbonate species in previous literature.[2,32,37]

Interestingly, a clear shoulder in the same position arises in the Cu sample at -0.2 V vs RHE only in the backward scan (**Figure S18a**), consistent with the previous literature studies showing Cu-carbonate formation upon returning to OCP.

We note that some dynamic changes in terms of peak shape and potential shifts occur at more negative potential for the carbonate bands and for the spectra more generally (e.g. -0.8 V vs RHE). These changes might be attributed to dynamicity of those adsorbed carbonate species as well of dynamicity of the catalytic surfaces. We also note that intensifying bubble formation at more negative potential might equally affect the signal. Future investigations are needed to assign the origin of this dynamicity observed as well as a more quantitative peak deconvolution.

Yet, these results indicate that Cu carbonate species indeed persist under cathodic bias on the copper surface when starting from Cu_{OCP}.

Additionally, we monitored the broad CO band at high Raman shift during the forward and backward potential ramp (**Figure S18d,e**). The reversibility of this band has been associated with Cu surface reconstruction.[32] We observe a quasi-reversible CO band in the case of Cu (**Figure S18d**). In contrast, the CO signal is rapidly lost for the Cu_{OCP} during the backward scan beginning at -0.6 V vs RHE (**Figure S18e**), which may correlate with the progressive deactivation observed for the Cu carbonate-rich samples (**Figure 2**).[32,37]

Table S2: Overview of the main vibrations observed during SERS measurement in this work with corresponding peak assignments

**Raman bands (cm^{-1})**	**Peak assignment**	**Ref.**
1540	ν_{as} (carbonate) on Cu	[2,32,33,37]
1570	ν_{as} (carboxylate) on Cu	[32,33,38]
1650-1675	δ(H–O–H) of interfacial water molecules	[36]
2090	ν(C-O) from adsorbed CO	[2,32,33,36,37]

References

(1) Iyengar, P.; Kolb, M. J.; Pankhurst, J. R.; Calle-Vallejo, F.; Buonsanti, R. Elucidating the Facet-Dependent Selectivity for CO2Electroreduction to Ethanol of Cu-Ag Tandem Catalysts. *ACS Catal* 2021, *11* (8), 4456–4463. https://doi.org/10.1021/acscatal.1c00420.

(2) Zhan, C.; Dattila, F.; Rettenmaier, C.; Bergmann, A.; Kühl, S.; García-Muelas, R.; López, N.; Roldan Cuenya, B. Revealing the CO Coverage-Driven C-C Coupling Mechanism for Electrochemical CO2Reduction on Cu2O Nanocubesvia OperandoRaman Spectroscopy. *ACS Catal* 2021, *11* (13), 7694–7701. https://doi.org/10.1021/acscatal.1c01478.

(3) Shen, T. H.; Girod, R.; Tileli, V. Insights into Electrocatalyst Transformations Studied in Real Time with Electrochemical Liquid-Phase Transmission Electron Microscopy. *Acc Chem Res* 2023, *56* (21), 3023–3032. https://doi.org/10.1021/acs.accounts.3c00463.

(4) Buchholz, T. O.; Krull, A.; Shahidi, R.; Pigino, G.; Jékely, G.; Jug, F. Content-Aware Image Restoration for Electron Microscopy. *Methods Cell Biol* 2019, *152*, 277–289. https://doi.org/10.1016/bs.mcb.2019.05.001.

(5) Hochfilzer, D.; Tiwari, A.; Clark, E. L.; Bjørnlund, A. S.; Maagaard, T.; Horch, S.; Seger, B.; Chorkendorff, I.; Kibsgaard, J. In Situ Analysis of the Facets of Cu-Based Electrocatalysts in Alkaline Media Using Pb Underpotential Deposition. *Langmuir* 2022, *38* (4), 1514–1521. https://doi.org/10.1021/acs.langmuir.1c02830.

(6) Albertini, P. P.; Calderon Mora, J.; Guskova, M.; Rodlamul, P.; Hicks, N.; Leemans, J.; Loiudice, A.; Buonsanti, R. Active Sites under Electronic Effect Are More Sensitive to Microenvironment in CO2 Electroreduction. *J Am Chem Soc* 2025. https://doi.org/10.1021/jacs.5c05697.

(7) Pankhurst, J. R.; Iyengar, P.; Loiudice, A.; Mensi, M.; Buonsanti, R. Metal-Ligand Bond Strength Determines the Fate of Organic Ligands on the Catalyst Surface during the Electrochemical CO2reduction Reaction. *Chem Sci* 2020, *11* (34), 9296–9302. https://doi.org/10.1039/d0sc03061a.

(8) Albertini, P. P.; Newton, M. A.; Wang, M.; Segura Lecina, O.; Green, P. B.; Stoian, D. C.; Oveisi, E.; Loiudice, A.; Buonsanti, R. Hybrid Oxide Coatings Generate Stable Cu

Catalysts for CO2 Electroreduction. *Nat Mater* 2024, *23* (5), 680–687. https://doi.org/10.1038/s41563-024-01819-x.

(9) Biesinger, M. C. Advanced Analysis of Copper X-Ray Photoelectron Spectra. *Surface and Interface Analysis* 2017, *49* (13), 1325–1334. https://doi.org/10.1002/sia.6239.

(10) Grosse, P.; Gao, D.; Scholten, F.; Sinev, I.; Mistry, H.; Roldan Cuenya, B. Dynamic Changes in the Structure, Chemical State and Catalytic Selectivity of Cu Nanocubes during CO2 Electroreduction: Size and Support Effects. *Angewandte Chemie* 2018, *130* (21), 6300–6305. https://doi.org/10.1002/ANGE.201802083.

(11) Grosse, P.; Yoon, A.; Rettenmaier, C.; Herzog, A.; Chee, S. W.; Roldan Cuenya, B. Dynamic Transformation of Cubic Copper Catalysts during CO2 Electroreduction and Its Impact on Catalytic Selectivity. *Nat Commun* 2021, *12* (1). https://doi.org/10.1038/s41467-021-26743-5.

(12) Timoshenko, J.; Bergmann, A.; Rettenmaier, C.; Herzog, A.; Arán-Ais, R. M.; Jeon, H. S.; Haase, F. T.; Hejral, U.; Grosse, P.; Kühl, S.; Davis, E. M.; Tian, J.; Magnussen, O.; Roldan Cuenya, B. Steering the Structure and Selectivity of CO2 Electroreduction Catalysts by Potential Pulses. *Nat Catal* 2022, *5* (4), 259–267. https://doi.org/10.1038/s41929-022-00760-z.

(13) Nitopi, S.; Bertheussen, E.; Scott, S. B.; Liu, X.; Engstfeld, A. K.; Horch, S.; Seger, B.; Stephens, I. E. L.; Chan, K.; Hahn, C.; Nørskov, J. K.; Jaramillo, T. F.; Chorkendorff, I. Progress and Perspectives of Electrochemical CO2 Reduction on Copper in Aqueous Electrolyte. *Chem Rev* 2019, *119* (12), 7610–7672. https://doi.org/10.1021/acs.chemrev.8b00705.

(14) Chen, Z.; Ye, Y.; Jiang, K. Metal–Metal Oxide Hybrid Catalysts for Electrocatalytic CO2 Reduction Reaction. *Chemical Physics Reviews* 2024, *5* (4). https://doi.org/10.1063/5.0223542.

(15) Li, C. W.; Kanan, M. W. CO 2 Reduction at Low Overpotential on Cu Electrodes Resulting from the Reduction of Thick Cu 2O Films. *J Am Chem Soc* 2012, *134* (17), 7231–7234. https://doi.org/10.1021/ja3010978.

(16) Ren, D.; Deng, Y.; Handoko, A. D.; Chen, C. S.; Malkhandi, S.; Yeo, B. S. Selective Electrochemical Reduction of Carbon Dioxide to Ethylene and Ethanol on Copper(I) Oxide Catalysts. *ACS Catal* 2015, *5* (5), 2814–2821. https://doi.org/10.1021/cs502128q.

(17) Lei, Q.; Zhu, H.; Song, K.; Wei, N.; Liu, L.; Zhang, D.; Yin, J.; Dong, X.; Yao, K.; Wang, N.; Li, X.; Davaasuren, B.; Wang, J.; Han, Y. Investigating the Origin of Enhanced C2+Selectivity in Oxide-/Hydroxide-Derived Copper Electrodes during CO2Electroreduction. *J Am Chem Soc* 2020, *142* (9), 4213–4222. https://doi.org/10.1021/jacs.9b11790.

(18) Long, C.; Liu, X.; Wan, K.; Jiang, Y.; An, P.; Yang, C.; Wu, G.; Wang, W.; Guo, J.; Li, L.; Pang, K.; Li, Q.; Cui, C.; Liu, S.; Tan, T.; Tang, Z. Regulating Reconstruction of Oxide-Derived Cu for Electrochemical CO2 Reduction toward n-Propanol. *Sci Adv* 2023, *9* (43). https://doi.org/10.1126/sciadv.adi6119.

(19) Lum, Y.; Yue, B.; Lobaccaro, P.; Bell, A. T.; Ager, J. W. Optimizing C-C Coupling on Oxide-Derived Copper Catalysts for Electrochemical CO2 Reduction. *Journal of Physical Chemistry C* 2017, *121* (26), 14191–14203. https://doi.org/10.1021/acs.jpcc.7b03673.

(20) Tang, W.; Peterson, A. A.; Varela, A. S.; Jovanov, Z. P.; Bech, L.; Durand, W. J.; Dahl, S.; Nørskov, J. K.; Chorkendorff, I. The Importance of Surface Morphology in Controlling the Selectivity of Polycrystalline Copper for CO2 Electroreduction. *Physical Chemistry Chemical Physics* 2012, *14* (1), 76–81. https://doi.org/10.1039/c1cp22700a.

(21) Ma, M.; Djanashvili, K.; Smith, W. A. Controllable Hydrocarbon Formation from the Electrochemical Reduction of CO2 over Cu Nanowire Arrays. *Angewandte Chemie - International Edition* 2016, *55* (23), 6680–6684. https://doi.org/10.1002/anie.201601282.

(22) Kas, R.; Kortlever, R.; Yilmaz, H.; Koper, M. T. M.; Mul, G. Manipulating the Hydrocarbon Selectivity of Copper Nanoparticles in CO2 Electroreduction by Process Conditions. *ChemElectroChem* 2015, *2* (3), 354–358. https://doi.org/10.1002/celc.201402373.

(23) Velasco-Vélez, J. J.; Jones, T.; Gao, D.; Carbonio, E.; Arrigo, R.; Hsu, C. J.; Huang, Y. C.; Dong, C. L.; Chen, J. M.; Lee, J. F.; Strasser, P.; Roldan Cuenya, B.; Schlögl, R.;

Knop-Gericke, A.; Chuang, C. H. The Role of the Copper Oxidation State in the Electrocatalytic Reduction of CO 2 into Valuable Hydrocarbons. *ACS Sustain Chem Eng* 2019, *7* (1), 1485–1492. https://doi.org/10.1021/acssuschemeng.8b05106.

(24) Asiri, A. M.; Gao, J.; Khan, S. B.; Alamry, K. A.; Marwani, H. M.; Khan, M. S. J.; Adeosun, W. A.; Zakeeruddin, S. M.; Ren, D.; Grätzel, M. Revisiting the Impact of Morphology and Oxidation State of Cu on CO2Reduction Using Electrochemical Flow Cell. *Journal of Physical Chemistry Letters* 2022, *13* (1), 345–351. https://doi.org/10.1021/acs.jpclett.1c03957.

(25) Lum, Y.; Ager, J. W. Stability of Residual Oxides in Oxide-Derived Copper Catalysts for Electrochemical CO2 Reduction Investigated with 18O Labeling. *Angewandte Chemie - International Edition* 2018, *57* (2), 551–554. https://doi.org/10.1002/anie.201710590.

(26) Loiudice, A.; Lobaccaro, P.; Kamali, E. A.; Thao, T.; Huang, B. H.; Ager, J. W.; Buonsanti, R. Tailoring Copper Nanocrystals towards C2 Products in Electrochemical CO2 Reduction. *Angewandte Chemie - International Edition* 2016, *55* (19), 5789–5792. https://doi.org/10.1002/anie.201601582.

(27) Leemans, J.; Calderon Mora, J.; Albertini, P. P.; Kumar, K.; Boulanger, C.; Buonsanti, R. The Chemical Nature of the Oxide Directs the Stability and Reactivity of Copper|Oxide Interfaces in the Electrochemical CO2 Reduction Reaction. *Chemistry of Materials* 2025. https://doi.org/10.1021/acs.chemmater.5c00135.

(28) Aitbekova, A.; Zhou, C.; Stone, M. L.; Lezama-Pacheco, J. S.; Yang, A. C.; Hoffman, A. S.; Goodman, E. D.; Huber, P.; Stebbins, J. F.; Bustillo, K. C.; Ercius, P.; Ciston, J.; Bare, S. R.; Plessow, P. N.; Cargnello, M. Templated Encapsulation of Platinum-Based Catalysts Promotes High-Temperature Stability to 1,100 °C. *Nat Mater* 2022, *21* (11), 1290–1297. https://doi.org/10.1038/s41563-022-01376-1.

(29) Liccardo, G.; Cendejas, M. C.; Mandal, S. C.; Stone, M. L.; Porter, S.; Nhan, B. T.; Kumar, A.; Smith, J.; Plessow, P. N.; Cegelski, L.; Osio-Norgaard, J.; Abild-Pedersen, F.; Chi, M.; Datye, A. K.; Bent, S. F.; Cargnello, M. Unveiling the Stability of Encapsulated Pt Catalysts Using Nanocrystals and Atomic Layer Deposition. *J Am Chem Soc* 2024, *146* (34), 23909–23922. https://doi.org/10.1021/jacs.4c06423.

(30) Sebastián-Pascual, P.; Escudero-Escribano, M. Surface Characterization of Copper Electrocatalysts by Lead Underpotential Deposition. *Journal of Electroanalytical Chemistry* 2021, *896* (May). https://doi.org/10.1016/j.jelechem.2021.115446.

(31) Bernasconi, F.; Plainpan, N.; Mirolo, M.; Wang, Q.; Zeng, P.; Battaglia, C.; Senocrate, A. Operando Observation of (Bi)Carbonate Precipitation during Electrochemical CO2 Reduction in Strongly Acidic Electrolytes. *ACS Catalysis* 2024, *14* (11), 8232–8237. https://doi.org/10.1021/acscatal.4c01884.

(32) Amirbeigiarab, R.; Tian, J.; Herzog, A.; Qiu, C.; Bergmann, A.; Roldan Cuenya, B.; Magnussen, O. M. Atomic-Scale Surface Restructuring of Copper Electrodes under CO2 Electroreduction Conditions. *Nat Catal* 2023, *6* (9), 837–846. https://doi.org/10.1038/s41929-023-01009-z.

(33) De Ruiter, J.; An, H.; Wu, L.; Gijsberg, Z.; Yang, S.; Hartman, T.; Weckhuysen, B. M.; Van Der Stam, W. Probing the Dynamics of Low-Overpotential CO2-to-CO Activation on Copper Electrodes with Time-Resolved Raman Spectroscopy. *J Am Chem Soc* 2022, *144* (33), 15047–15058. https://doi.org/10.1021/jacs.2c03172.

(34) Moradzaman, M.; Mul, G. In Situ Raman Study of Potential-Dependent Surface Adsorbed Carbonate, CO, OH, and C Species on Cu Electrodes During Electrochemical Reduction of CO2. *ChemElectroChem* 2021, *8* (8), 1478–1485. https://doi.org/10.1002/celc.202001598.

(35) Rüscher, M.; Herzog, A.; Timoshenko, J.; Jeon, H. S.; Frandsen, W.; Kühl, S.; Roldan Cuenya, B. Tracking Heterogeneous Structural Motifs and the Redox Behaviour of Copper-Zinc Nanocatalysts for the Electrocatalytic CO2 Reduction Using Operando Time Resolved Spectroscopy and Machine Learning. *Catal Sci Technol* 2022, *12* (9), 3028–3043. https://doi.org/10.1039/d2cy00227b.

(36) Zhou, Y. W.; Ibáñez-Alé, E.; López, N.; Roldan Cuenya, B.; Kley, C. S. Carbonate Anions and Radicals Induce Interfacial Water Ordering in CO2 Electroreduction on Gold. *Nat Chem* 2025. https://doi.org/10.1038/s41557-025-01977-8.

(37) Jiang, S.; Klingan, K.; Pasquini, C.; Dau, H. New Aspects of Operando Raman Spectroscopy Applied to Electrochemical CO2 Reduction on Cu Foams. *Journal of Chemical Physics* 2019, *150* (4). https://doi.org/10.1063/1.5054109.

(38) Chernyshova, I. V.; Somasundaran, P.; Ponnurangam, S. On the Origin of the Elusive First Intermediate of CO2 Electroreduction. *Proc Natl Acad Sci U S A* 2018, *115* (40), E9261–E9270. https://doi.org/10.1073/pnas.1802256115.
